# Diversity and Diversification: Ecosystem Services Derived From Underutilized Crops and Their Co-benefits for Sustainable Agricultural Landscapes and Resilient Food Systems in Africa

**DOI:** 10.3389/fagro.2022.859223

**Published:** 2022-05-04

**Authors:** Tafadzwanashe Mabhaudhi, Sithabile Hlahla, Vimbayi Grace Petrova Chimonyo, Rebecka Henriksson, Tendai Polite Chibarabada, Vongai G. Murugani, Vivienne P. Groner, Zerihun Tadele, Nafiisa Sobratee, Rob Slotow, Albert Thembinkosi Modi, Frédéric Baudron, Pauline Chivenge

**Affiliations:** 1 Centre for Transformative Agricultural and Food Systems, School of Agricultural, Earth and Environmental Sciences, University of KwaZulu-Natal, Pietermaritzburg, South Africa; 2 International Water Management Institute-Ghana (IWMI-GH), West Africa Office, c/o CSIR, Accra, Ghana; 3 Kwame Nkrumah University of Science and Technology (KNUST), Kumasi, Ghana; 4 Future Water Research Institute, University of Cape Town, Cape Town, South Africa; 5 International Maize and Wheat Improvement Center (CIMMYT)-Zimbabwe, Harare, Zimbabwe; 6 Centre for Water Resources Research, University of KwaZulu-Natal, Pietermaritzburg, South Africa; 7 Stockholm International Peace Research Institute, Stockholm, Sweden; 8 Centre for Biodiversity and Environment Research, Department of Genetics, Evolution and Environment, University College London, London, United Kingdom; 9 Institute of Plant Sciences, University of Bern, Bern, Switzerland; 10 Centre for Transformative Agricultural and Food Systems, School of Life Sciences, University of KwaZulu-Natal, Pietermaritzburg, South Africa; 11 Department of Genetics, Evolution and Environment, University College London, London, United Kingdom; 12 African Plant Nutrition Institute, UM6P Experimental Farm, Benguérir, Morocco

**Keywords:** biodiversity, food and nutrition security (FNS), poverty alleviation, ecosystem, sustainable livelihoods, transformative adaptation

## Abstract

There are growing calls to adopt more sustainable forms of agriculture that balance the need to increase production with environmental, human health, and wellbeing concerns. Part of this conversation has included a debate on promoting and mainstreaming neglected and underutilized crop species (NUS) because they represent a more ecologically friendly type of agriculture. We conducted a systematic review to determine the ecosystem services derived from NUS and assess their potential to promote functional ecological diversity, food and nutritional security, and transition to more equitable, inclusive, sustainable and resilient agricultural landscapes and food systems in Africa. Our literature search yielded 35 articles for further analysis. The review showed that NUS provide various provisioning, regulating, cultural, and supporting ecosystem services and several environmental and health co-benefits, dietary diversity, income, sustainable livelihood outcomes, and economic empowerment, especially for women. Importantly, NUS address the three pillars of sustainable development-ecological, social, and economic. Thus, NUS may provide a sustainable, fit-for-purpose transformative ecosystem-based adaptation solution for Africa to transition to more sustainable, healthy, equitable, and resilient agricultural landscapes and food systems.

## Introduction

There is consensus that current food systems need to be transformed to be more resilient, sustainable, and equitable for the attainment of food and nutritional security (Caron et al., 2018; FAO, 2018; The Economics of Ecosystems Biodiversity, 2018). The intensification of global food production is taking a toll on environmental resources (Caron et al., 2018; IPBES, 2018; Béné et al., 2020), impacting biodiversity and ecosystem services and functioning (IPBES, 2018; Borelli et al., 2020). Ecosystem services are defined by the Millennium Ecosystem Assessment (2005, 40) as the “benefits people obtain from ecosystems,” namely provisioning, regulating, and cultural services, that directly affect people and the supporting services needed to maintain other services. Ecosystem services are vital for sustaining human wellbeing and activities and future economic and social development (The Economics of Ecosystems Biodiversity, 2008; IPBES, 2018; Wood et al., 2018; Borelli et al., 2020). Therefore, we need to enhance ecosystem services as part of strategies to transition toward more sustainable agricultural landscapes and food systems that can achieve sustainable human health and wellbeing, social, ecological, and economic outcomes; this calls for transformative approaches. Diversifying agricultural landscapes, food systems and diets with neglected and underutilized crop species (NUS) may be one way to facilitate such transformation (Borelli et al., 2020).

Neglected and underutilized species are described as non-commodity wild or cultivated plant species, including crop wild relatives, that were once popular but have since been neglected due to a range of agronomic, genetic, economic, social, and cultural reasons (Padulosi and Hoeschle-Zeledon, 2004; Kasolo et al., 2018). Several authors have advocated for the inclusion of NUS in global diets as part of a transition to sustainable and healthy food systems (Mayes et al., 2012; Chivenge et al., 2015; Baldermann et al., 2016; Borelli et al., 2020), despite the fact that they are generally low yielding (Mabhaudhi et al., 2017b). This is premised on reports that NUS: (i) are nutrient-dense, locally available and affordable sources of macro- and micro-nutrients such as calcium, vitamins, iron, zinc, ascorbic acid, provitamin A carotenoids, and folic acid (Aworh, 2018); (ii) are suited to low input type agriculture (i.e., they do not require significant use of fertilizers, herbicides, pesticides, and other agricultural inputs, and thrive with little care; Aworh, 2018; Kasolo et al., 2018); (iii) possess tolerance to several abiotic and biotic stresses such as drought, heat, and salinity stress, as well as to pests and diseases (Jena et al., 2018; Tadele, 2018; Borelli et al., 2020); (iv) offer ecologically friendly ways of intensifying agricultural production (Mayes et al., 2012; Padulosi et al., 2013; Aworh, 2018), especially under water scarcity (Baldermann et al., 2016; Mabhaudhi et al., 2019); (v) provide health and environmental co-benefits due to their high nutrient content, medicinal properties, and contribution to biodiversity (Baldermann et al., 2016; Kasolo et al., 2018); and (vi) are genetically diverse, and can potentially contribute to biodiversity conservation and the generation of ecosystem services (Chivenge et al., 2015; Zhang et al., 2019).

We hypothesized that given the environmental and social unsustainability of the current global food system (Mayes et al., 2012; Baa-Poku, 2018; FAO, 2018), and the high levels of malnutrition globally (Mayes et al., 2012; Baldermann et al., 2016; FAO, 2019), wider use of NUS could contribute to more sustainable, equitable and resilient agricultural landscapes and food systems. This can be achieved by diversifying monocrop agricultural systems with NUS, which can contribute to nature-based transformative ecosystem-based adaptation efforts in agriculture, sustainable livelihood outcomes, and good quality of life for all (Díaz et al., 2018). The study’s objective was to better understand these benefits from a resilience and sustainability perspective to identify the range of ecosystem services NUS provide in Africa, using a systematic literature review. Moreover, very few policies in Africa, multi-actor and/or multi-sector, address or incorporate NUS. This study also aims to address this policy gap. Africa was chosen due to the importance of NUS to food, nutrition, and culture on the continent (Mayes et al., 2012; Baa-Poku, 2018; Kasolo et al., 2018) and the increasing NUS-related research that is being conducted within African countries. Moreover, the continent experiences various socio-economic and developmental challenges, such as high poverty levels, malnutrition, inequality, rapid urbanization, and economic decline (GNFC and FSIN, 2020).

## Methodology

We adopted a two-phased approach that included a systematic literature review and systemic analyses in determining the ecosystem services provided by neglected and underutilized species (NUS) in Africa. The preconceived themes or ecosystem services categories were derived from the Economics of Ecosystems and Biodiversity (TEEB) framework, a global initiative with the goals of evaluating nature (The Economics of Ecosystems Biodiversity, 2008; TEEB, 2010). The categories were provisioning, regulating, habitat and support, and cultural services. TEEB derives from the Millennium Ecosystem Assessment (MEA; The Economics of Ecosystems Biodiversity, 2008). The initiative modified the ecosystem categories that the MEA provided to address the link between biodiversity and ecosystem services (Evers et al., 2018). Details of the TEEB are provided under Supplementary Material 1.

### Phase I: Systematic Review

A systematic literature review was conducted using the SCOPUS database, one of the largest scientific databases of peer-reviewed literature. The reporting model provided by PRISMA (Preferred Reporting Items for Systematic Reviews and Meta-Analyses) was used (Moher et al., 2009). The PRISMA checklist (http://www.prisma-statement.org/) was used as a guideline to avoid biased reporting (Moher et al., 2015; Shamseer et al., 2015). Within the database, search terms were set to be in the title, abstract and keywords to avoid retrieval of articles where the search terms only appear in the reference lists of full-text articles. The search terms included the following:

• (“Neglected AND underutilized” OR “Neglected” OR “Underutilized” OR “Orphan”) AND (“Crop*” OR “Species” OR “Tree*”) AND (“Ecosystem Service*”)
• (“Neglected AND underutilized” OR “Neglected” OR “Underutilized” OR “Orphan”) AND (“Crop*” OR “Species” OR “Tree*”) AND (“Provisioning Service*”)
• (“Neglected AND underutilized” OR “Neglected” OR “Underutilized” OR “Orphan”) AND (“Crop*” OR “Species” OR “Tree*”) AND (“Regulating Service*”)
• (“Neglected AND underutilized” OR “Neglected” OR “Underutilized” OR “Orphan”) AND (“Crop*” OR “Species” OR “Tree*”) AND (“Habitat and Support Service*”)
• (“Neglected AND underutilized” OR “Neglected” OR “Underutilized” OR “Orphan”) AND (“Crop*” OR “Species” OR “Tree*”) AND (“Cultural Service*”)

Additional searches were conducted using examples of the types of ecosystem services provided, bringing the total number of searches conducted to 44. A list of the exact search strings used in SCOPUS is provided in Supplementary Material 3.

During the initial search, the search terms yielded 27,848 hits. The titles, keywords, and abstracts were screened within the SCOPUS database, and 366 articles were found to focus on both ecosystems services and NUS (see Supplementary Table 3). These were considered for further screening. These articles’ titles, keywords, and abstracts were exported to Endnote reference manager to remove 104 duplicates (see Supplementary Tables 4, 5). Inclusion and exclusion criteria were then applied to the abstracts of the remaining 262 articles in Microsoft Excel, leaving 123 articles. The inclusion criteria for the search were articles referring to food provision. In contrast, the exclusion criteria were articles not in English, books or book chapters, or articles focusing on animals and invertebrates and non-food crops. Books and book chapters were excluded due to the focus of the study on NUS research in the academic field in the peer-reviewed literature.

**Figure 1:**
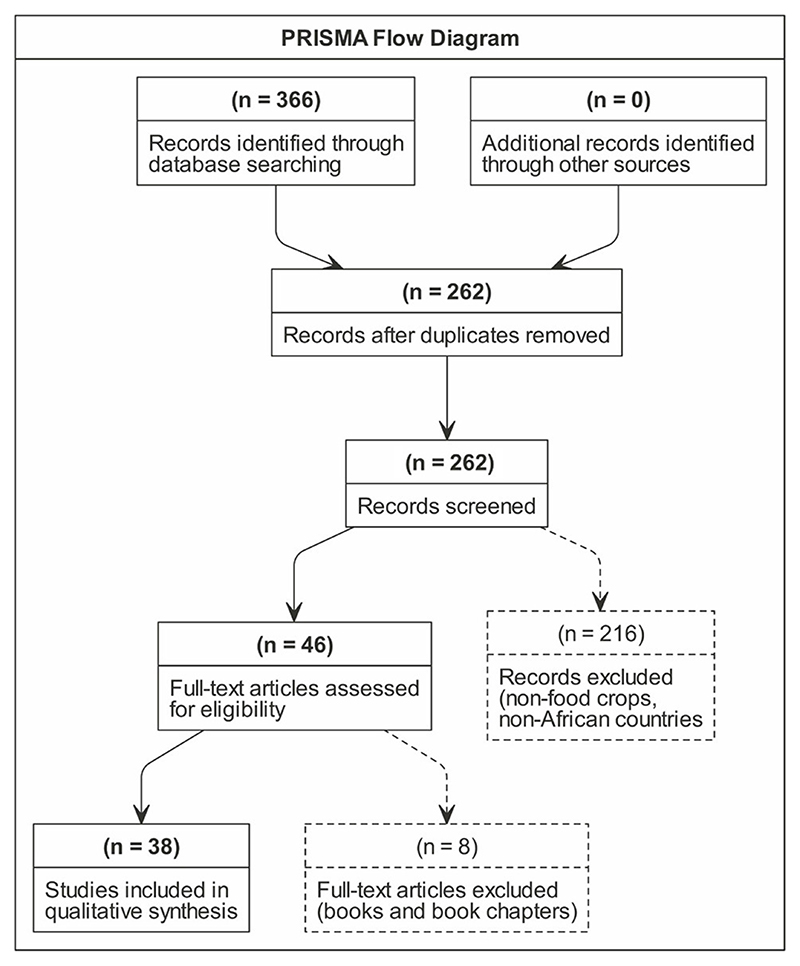
PRISMA flow diagram.

Further screening of the 123 articles revealed that 46 focused on Africa. The full texts of these were retrieved and assessed for eligibility, and a further 11 books and book chapters were removed, leaving 35 articles (see Figure 1 and Supplementary Table 5). A thematic deductive analysis was then undertaken on the 35 full-text articles to synthesize the findings and establish the kinds of ecosystem services provided by the NUS discussed in the articles. Thematic deductive analysis is defined as coding the data based on a pre-existing coding framework or the researcher’s analytic preconceptions (Braun and Clarke, 2006; Nowell et al., 2017). This study used a deductive method to code the ecosystem services. The analysis was based on the ecosystem services listed in TEEB.

### Phase II: Seeking a Systemic Understanding to Embed NUS to Sustainable Agricultural Landscapes and Food Systems in Africa

Two systems diagrams were developed to combine causal loop diagramming (CLD) and stock accumulation to demonstrate a systemic structure causing the delayed transitioning of the smallscale agricultural landscape toward sustainable and resilient food systems. Systems thinking is a holistic approach to analysis that focuses on how a system’s constituent parts interrelate and how systems work over time and within the context of larger systems (Reynolds and Holwell, 2010). Systems archetypes have been applied in agricultural and conservation management to clarify complex, interrelated factors (Sharif and Irani, 2016; Hallett, 2020; Nyam et al., 2020).

In the present work, first, the “Shifting the burden” archetype was used to link the fact that modern and monoculture agriculture has acted as a symptomatic solution to developing small-scale agriculture and food system in Africa and that a more fundamental approach to transform and diversify cropping systems for improved resilience is needed. Second, the transitioning process is expressed in its components, ecosystems services and the expected and/or potential cobenefits. Therefore, the ecosystem services provided by the NUS crops would be forming part of wider social and environmental systems. It is envisioned that the dynamics of the interrelated subsystems would address overarching United Nations Sustainable Development Goals altogether.

Systems thinking starts with understanding the problems under focus and its fundamental system structure, that is, recognizing interconnections and understanding feedback. In the CLD, arrows show the influence of one variable on another—a change in the cause leads to a change in the effect (Reynolds and Holwell, 2010). The polarity of the arrows indicates the factual relationship between any two nodes, which illustrates the causal link. A simple stock and flow network is also used to depict accumulation and the corresponding rate of change over time (Reynolds and Holwell, 2010). The interplay of feedback loops gives rise to a realistic multi-loop system that explains behavior through time (Hallett, 2020).

## Results

### Literature Search

Following the systematic search, 35 articles were selected for further analysis. These articles referred to a total of 218 foodbased NUS (see Supplementary Table 6). Fifty-one percent of the NUS were fruit trees and shrubs, 35% were leafy vegetables, herbs, and vegetables, 6% were legumes, and 4% were cereals (Supplementary Figure 1).

Thirty-one publications discussed the provisioning services provided by NUS. A majority (29) discussed how NUS are a sustainable source of food and nutrition; 11 indicated that they are a source of raw materials, and 16 stated that they are a source of medicine (traditional or pharmaceutical). Eight articles stated they could provide feed and fodder for livestock (Supplementary Table 7). Twenty-three articles in the study discussed regulating services, with the majority (13) mentioning climate regulation, seven mentioned water regulation, six discussed moderation of extreme events, and three discussed pollination. Three mentioned erosion prevention and soil fertility (Supplementary Table 7). Fourteen articles discussed habitat and support, with all 14 highlighting the genetic diversity of NUS. Eighteen articles discussed the cultural services provided by NUS. Of these 18 articles, a majority (12) referred to indigenous knowledge, seven mentioned cultural heritage, four mentioned spiritual/ religious sense of place, and four discussed eco- and cultural tourism (Supplementary Table 7).

### Ecosystem Services Provided by NUS

**Figure 2:**
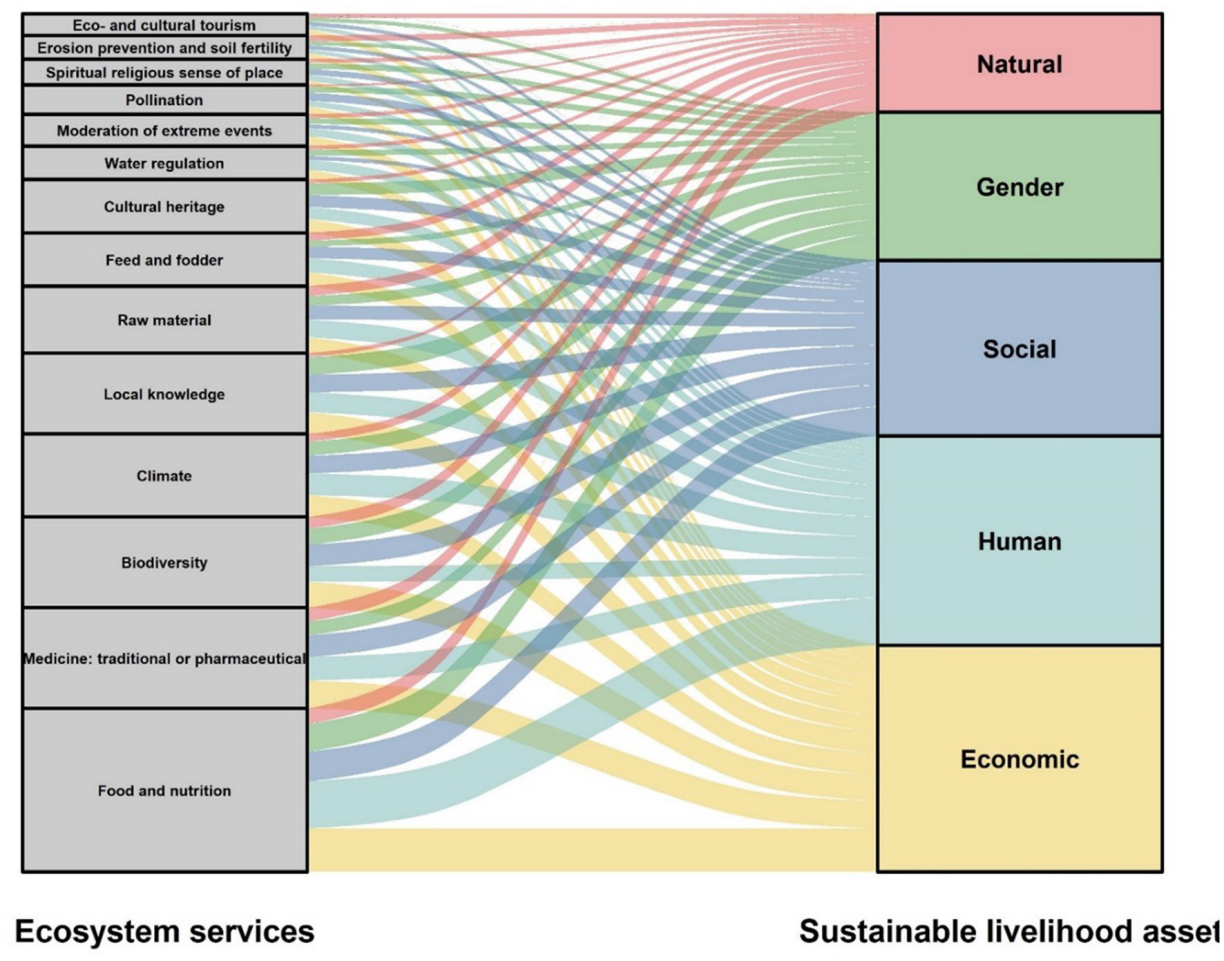
Potential contribution of ecosystem services (The Economics of Ecosystems Biodiversity, 2008) provided by NUS to the provision of sustainable livelihood assets for marginalized groups, based on literature. The height of a block is proportional to the number of ecosystem services included in the cluster, and the width of a stream field is proportional to the number of ecosystem services included in both clusters connected by the stream.

The research revealed that NUS could provide provisioning, regulating, cultural, habitat and support services; these are provided in Supplementary Table 7 and below (Figure 2).

#### Provisioning Services

Provisioning services refer to ecosystems’ material or energy outputs, such as food, fiber, and water (Millennium Ecosystem Assessment, 2005; The Economics of Ecosystems Biodiversity, 2018). NUS provides food and nutrition, raw materials, medicine (traditional or pharmaceutical), and feed and fodder (Supplementary Figure 1 and Supplementary Table 7). It was widely agreed that NUS are high in macro and micronutrients, in particular, potassium, calcium, magnesium, and zinc, and vitamins such as K, C, E, and folate, essential oils and bioactive compounds such as flavonoids, antioxidants, phenolics, and carotenoids (Johns and Eyzaguirre, 2006; Liu et al., 2011; Hughes and Ebert, 2013; Nyadanu and Lowor, 2014; Masondo et al., 2016; Tele Ayenan and Ezin, 2016; Nitcheu Ngemakwe et al., 2017; Campanaro et al., 2019), all of which are important for nutrition security and human and livestock health (see Table 1). Nyamupingidza et al. (2013) noted that the nutritional and chemical diversity of NUS makes them ideal nutritional supplements in diets of people with compromised immune systems (e.g., HIV/AIDS), reduces the incidence of non-communicable diseases (NCDs), improves wellbeing, and increases longevity (Table 1). This is because they are high in macronutrients such as carbohydrates, proteins, fats, and inorganic constituents and have anti-diarrhoeal, anti-diabetic, antiplasmodial, antibacterial, and antiviral properties (Masondo et al., 2016). For example, Kersting’s groundnut *(Macrotyloma geocarpum)* reduces the occurrence of atherosclerosis and coronary heart disease, as it has a low fat (1.0%) and sodium content (5.67mg g^−1^; Ayenan and Ezin, 2016).These health benefits provided by NUS are inexpensive and readily available, especially for the poor.

Despite the numerous favorable properties of NUS, the research revealed that they have a few negative attributes. The leaves of false sesame *(Ceratotheca sesamoides)* are bitter and have a laxative effect, prompting consumers to find ways of preparation to reduce these effects (Masondo et al., 2016). These preparation methods include the addition of onions and tomatoes to soften and reduce the bitterness of *C. sesamoides’* leaves or serve with other vegetables to reduce the laxative effect (Masondo et al., 2016). Another problem arises from the processing and consumption of gari (roasted cassava granule; Manihot esculenta). The processing stage has two major impacts on water resources: (i) the abstraction of processing water from surface and/or groundwater (i.e., blue water), and (ii) pollution of water due to waste flows from the cassava processing sites (Adeoti et al., 2009). Waste flows contain soluble cyanide and organic acid, and fine particles of grated mash (Adeoti et al., 2009). During consumption, there is the abstraction of water from surface and sub-surface sources at the household level for the processing of gari, either as a snack or as dough-like paste, as well as waste flows (mostly organic) resulting from the cleaning of utensils used in the consumption process (Adeoti et al., 2009). This can result in excessive use of scarce water resources and water pollution. It has been established that the processing and consumption of gari may bear negative consequences for water resources due to the reasons stated above.

#### Regulating Services

Regulating services are the benefits obtained from regulating ecosystem processes such as the regulation of climate or air quality (Millennium Ecosystem Assessment, 2005). Intensive agriculture has led to increased food production but at the cost of regulating services (IPBES, 2018). IPBES (2018) projected that regulating services will continue to decline as global demand for provisioning services such as food, feed, timber, and bioenergy increases. NUS can contribute to an environmentally beneficial and inexpensive solution by contributing to regulating services. Our study revealed that NUS have the potential to maintain biodiversity in agriculture; this provides regulatory ES such as nutrient cycling, carbon sequestration, soil erosion control, reduction of greenhouse gases (GHGs), and hydrological control processes (Chivenge et al., 2015; Tele Ayenan and Ezin, 2016; Table 1).

Leguminous NUS can fix atmospheric nitrogen and mobilize nutrients from the subsoil (Chivenge et al., 2015; Tele Ayenan and Ezin, 2016; Table 1). For example, Kersting’s groundnut can fix up to 16.5–57.8kg ha^−1^ of atmospheric N_2_ (Tele Ayenan and Ezin, 2016). This function is particularly crucial for resource-constrained farmers who cannot afford to purchase inputs such as inorganic nitrogen fertilizers (Chivenge et al., 2015) and rehabilitate degraded agro-ecosystems (Tele Ayenan and Ezin, 2016). It is also important to acknowledge that NUS has trade-offs given their general low productivity compared to intensive monocrop systems (Mabhaudhi et al., 2017b). However, given the low fertilizer use in Africa (smallholder), an argument can be made that NUS may produce more within marginal environments.

Since NUS are locally adapted to the environments in which they grow and have a lower carbon footprint than most major staple crops, growing them may cause less land and ecosystem disturbance. This will contribute to conserving local biodiversity and overall ecosystem functioning (Mabhaudhi et al., 2019). Mabhaudhi et al. (2019) also estimate that transitioning to more plant-based food systems, including NUS, could reduce food-related greenhouse gas emissions by ~29–70%. The point to note is that NUS represents a more natural, undisturbed agroecosystem that supports natural systems in providing ecosystem services and reduces trade-offs associated with ecosystem services in monocropped agricultural systems.

Many NUS are less susceptible to pests and diseases than many common crops (Table 1), and therefore, require fewer pesticide and fertilizer interventions (Hughes and Ebert, 2013; Mabhaudhi et al., 2019), and can adapt to different agro-ecological environments (Karunaratne et al., 2015). For example, indigenous vegetable melon *(Cucumis melo subsp. Agrestis)* is resistant to several pests, including leaf miners, fruit flies, and diseases such as Fusarium wilt *(Fusarium oxysporum)*, powdery mildew, Zucchini yellow mosaic virus (*Potyvirus*), Cucurbit aphid-borne yellows virus (*Polerovirus*; Hughes and Ebert, 2013). Due to their low requirement and use of agrochemicals, NUS can mitigate the negative impacts that agrochemicals have on the environment, promote biodiversity, and maintain ecosystem health (Mabhaudhi et al., 2019). However, much of this remains anecdotal as there is still a lack of empirical data confirming most of these attributes.

#### Habitat and Support

Habitat and support services underpin all other ecosystem services necessary for production (Millennium Ecosystem Assessment, 2005). Unlike provisioning, regulating, and cultural services, the impacts of habitat and support services on people are indirect and long-term (Millennium Ecosystem Assessment, 2005). TEEB refers to two habitat and support services: habitat for species and maintenance of genetic diversity. The literature revealed that NUS could contribute to the latter, as the species are reported to be genetically diverse, and incorporating them into cropping systems can increase agricultural biodiversity in these systems (Quartermain, 2007; Gotor and Irungu, 2010; Hughes and Ebert, 2013; Rudebjer et al., 2013; Nyadanu and Lowor, 2014; Chivenge et al., 2015; Karunaratne et al., 2015; Masondo et al., 2016; Nyadanu et al., 2016; Tele Ayenan and Ezin, 2016; Mabhaudhi et al., 2017b; Towns and Shackleton, 2018; Campanaro et al., 2019). The agricultural biodiversity can increase the amount and type of wildlife species occupying the ecosystem (Chivenge et al., 2015). It can enable the ecosystem and food system to become more resilient to biotic and abiotic stressors (Chivenge et al., 2015; Nyadanu et al., 2016). The reduced use of agrochemicals also promotes biodiversity and beneficial biota contributing to overall ecosystem functioning.

#### Cultural Services

Neglected and underutilized species, especially traditional vegetables, are an essential part of Africa’s cultural heritage and identity and play important roles in customs and traditions (Dinssa et al., 2015; see Table 1). NUS farming practices are also embodied within cultural services, and these practices vary between species, households, and ethnic groups. For example, Buffalo thorn (*Ziziphus mucronata*) is used in connection with burial rites by the Zulu and Swazi tribes (Mokgolodi et al., 2011).

It is also believed to be immune to lightning (Mokgolodi et al., 2011) Anyone sheltering under the crop in a storm is considered to be safe (Mokgolodi et al., 2011). Farmers in Benin have traditional cultural farming practices that they apply to the traditional leafy vegetables, *Sesamum radiatum* and *Justicia tenella* (Etèka et al., 2011). These farming practices are utilized during seed collection; conservation, and germination (nursery handling and management); planting period and materials, space between individual plants; use of pesticides and fertilizers; harvest; production system; knowledge of pests and diseases; and the traditional methods of their control (Etèka et al., 2011). As we seek to transform agriculture and food systems toward greater sustainability and equity, preserving respect and promoting these values becomes crucial. This provides a pathway toward restoring indigenous people’s environmental rights and dignity.

#### Added-Value of Ecosystem Services Provided by NUS for Human Outcomes

##### Income Generation Opportunities

The current study revealed that products of NUS provisioning service could contribute toward household food security and be sold to supplement household income, thereby providing safety nets and improving the quality of life (Johns and Eyzaguirre, 2006; Quartermain, 2007; Gotor and Irungu, 2010; Etèka et al., 2011; Hughes and Ebert, 2013; Onyango et al., 2013; Rudebjer et al., 2013; Tebkew et al., 2014; Chivenge et al., 2015; Dinssa et al., 2015; Tele Ayenan and Ezin, 2016; Mabhaudhi et al., 2017b, 2019; Nitcheu Ngemakwe et al., 2017). This could be achieved with minimal resource inputs and pressure on land and water (Hughes and Ebert, 2013; Chivenge et al., 2015; Tele Ayenan and Ezin, 2016; Towns and Shackleton, 2018). Therefore, NUS can contribute to agricultural livelihoods, income diversification and help to reduce farmer vulnerability to environmental shocks (Mabhaudhi et al., 2019).

Neglected and underutilized crops such as worowo (*Solanecio biafrae)*, cockscomb *(Celosia argentea)*, amaranth and African eggplant *(Solanum macrocarpon)* can be cultivated with minimal capital investment and sold at a profit (Hughes and Ebert, 2013). In Benin, Kersting’s groundnut has a high economic value; it is up to five times the price of rice (Tele Ayenan and Ezin, 2016). This makes it an essential source of income for rural populations, as the average gross revenue earned is $1,200 per hectare (Tele Ayenan and Ezin, 2016). In such instances, high-value NUS can create economic opportunities for the local communities. Despite this, few formal markets are dedicated to NUS, and they are commonly sold in informal markets (Weinberger, 2007; Mabhaudhi et al., 2017b, 2019). However, where markets do exist, they are relatively easy to enter with low entry costs (Hughes and Ebert, 2013). Mabhaudhi et al. (2019) assert that NUS could provide rural smallholder farmers and women with an opportunity to participate in the agro-industrial food system by creating new employment, market, and distribution opportunities.

##### Role of Women in NUS Production

Women play a significant role in the production and promotion of NUS (Johns and Eyzaguirre, 2006; Gotor and Irungu, 2010; Hughes and Ebert, 2013; Rudebjer et al., 2013; Nyadanu and Lowor, 2014; Nyadanu et al., 2016; Mabhaudhi et al., 2017b, 2019). Gotor and Irungu (2010) report that women in Kenya are the main actors in producing and marketing African leafy vegetables. This role is not only crucial for the economic empowerment of women in rural areas; it can promote gender equality and assist in maintaining equity between female and male farmers if value chains are established (Hughes and Ebert, 2013; Dinssa et al., 2015). Johns and Eyzaguirre (2006, p. 184) also note that “knowledge and practice on the values and uses of agrobiodiversity in food systems is gendered, with women having greater knowledge about nutritional benefits and food uses of traditional species.” Mabhaudhi et al. (2019) concur that women are the main actors in NUS conservation, production, processing, marketing, and consumption, and they possess indigenous knowledge regarding the crops. As a result, NUS not only contribute toward zero hunger (SDG 2) of the sustainable development goals (SDGs), but no poverty (SDG 2), good health and wellbeing (SDG 3), and gender equality (SDG 5), as well. Furthermore, given the vulnerability of women and children to nutrition deficiencies in Africa (Johns and Eyzaguirre, 2006), NUS has the potential to ensure that these vulnerable groups can meet a significant portion of their daily nutritional needs.

## Discussion

**Figure 3:**
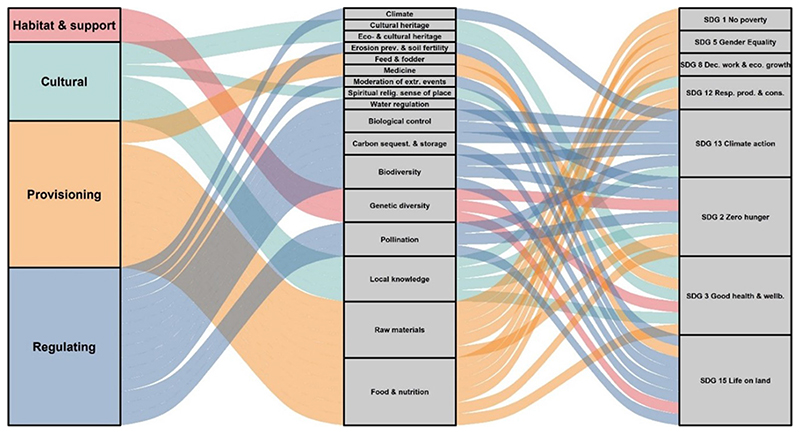
Perceived contribution of ecosystem services provided by NUS to the SDGs: Based on our findings, we deduced that NUS have the potential to contribute to the fulfillment of eight SDGs. The height of a block is proportional to the number of ecosystem services included in the cluster, and the width of a stream field is proportional to the number of ecosystem services included in both clusters connected by the stream.

In this section, we focus on how the agro-ecological, technological, environmental and socio-economic benefits of NUS and the mitigation co-benefits from the provisioning of ecosystem services can contribute to sustainable livelihoods (Figure 2); the 2030 Sustainable Development Goals (Figure 3); more sustainable, equitable and resilient agricultural and food systems; and other socio-ecological outcomes. The policy implications of NUS research in Africa and South Africa, in particular, are also discussed. The objective is not to replace staple crops with NUS. Rather, we seek to argue that including NUS within such existing systems and in-between successive sequences of major staple crops can enhance the overall resilience of the food system while increasing food productivity

### Sustainable Livelihood Outcomes

One of the significant benefits of NUS is the potential to enhance sustainable livelihoods within rural resource-poor households. This is particularly important in many African countries, which continue to struggle with high levels of poverty and inequality. Using the sustainable livelihood assets (SLAs) framework (Serrat, 2017), we identified interlinkages that exist between the reported ecosystem services provided by NUS and four SLAs, namely human, natural, social, and economic capital (Figure 2).

NUS provide vegetation coverage within natural systems and landscapes that offer an opportunity to replenish the natural resource base from which we derive ecosystem services and contribute to biodiversity (Chivenge et al., 2015; Tele Ayenan and Ezin, 2016). NUS diversify natural assets within cultivated arable lands by diversifying crop production systems, especially monoculture systems. The contribution to agro-biodiversity ensures that households have increased access to diverse capital streams, especially given the seasonality of staple crops (Ambrose-Oji, 2009). These crops can be sold for additional income, increasing financial (economic) capital (Figure 2), and also contribute to offsetting the cost of food, enabling communities to acquire physical assets or save funds, which may be redirected into autonomous pathways out of poverty (Ambrose-Oji, 2009). Furthermore, given the seasonal differences in the availability of common crops and climate change impacts, a combination of NUS and common crops can ensure a year-round supply of food and avoid nutritional deficits that typically occur during agricultural cycles (Ambrose-Oji, 2009). This can help reduce the vulnerability of livelihoods and build resilience, especially in rural areas, where there is a high dependence on agriculture and natural resource-based livelihood capital (Khatiwada et al., 2018).

The services provided by NUS, in particular, food and nutrition (Figure 2), also contribute toward human capital by improving human health and wellbeing, which enhances people’s ability and capacity to learn, engage in productive work, earn income and contribute to socio-economic development. NUS also contribute to social capital as communities can form networks to share indigenous knowledge regarding NUS and sell the crops (horizontal connections). This strengthens relationships between families and their neighbors by sharing the crops and provides an opportunity to use knowledge and skills (Ambrose-Oji, 2009; Balasha et al., 2019). This social cohesion is particularly beneficial in urban areas that lost social networks during rural-urban migration.

Urban and peri-urban agricultural activity can help recreate elements of rural environments, which are important for maintaining cultural identity and diversity, and the development of self-worth while engendering a sense of community and providing enjoyment and mental wellbeing (Ambrose-Oji, 2009). Therefore, if properly managed, the integration of NUS into urban home gardens can potentially contribute to the food supply in cities and enable the marginalized to make a living that is economically, ecologically, and socially sustainable (Krantz, 2001); this will improve the quality of life for many (Balasha et al., 2019).

### Sustainable Development Goals

Achieving the SDGs will require, among other things, judicious management of ecosystems and the protection of nature to ensure a sustainable flow of benefits and services to people (Wood et al., 2018). These services, and their contribution to the quality of life, are instrumental in the realization of the majority of the SDGs (Wood et al., 2018). NUS’s benefits can contribute to the fulfillment of several SDGs, with the provision of food and nutrition contributing to seven of these goals (Figure 3).

The crops offer an opportunity to earn income and escape poverty (SDG 1), provide nutrient-dense food, promote food and nutrition security, and sustainable agriculture (SDG 2), which can promote good health and wellbeing and reduce the incidence of NCDs (SDG 3). The medicinal properties of some of these crops can also promote health and wellbeing in marginalized areas where access to clinics and hospitals is often limited. Therefore, including NUS in diets can increase access to healthy diets, especially for poor and vulnerable groups, and decrease the hidden diet-related health costs associated with the current food consumption patterns (FAO, 2019). Furthermore, if value chains are created for NUS, they can be a source of employment and inclusive and sustainable local economic development (SDG 8). In addition, if properly developed, these value chains can promote gender equality as women are primarily involved in their production, which can empower them (SDG 5).

Sustainable agriculture has a central role to play in responsible consumption and production (SDG 12), climate change adaptation and mitigation (SDG 13), and preserving life on land (SDGG 15; Brooks, 2016). Given the potential role of NUS in making agricultural systems more resilient and sustainable, they, too, can contribute toward the fulfillment of these SDGs. In particular, the integration of NUS into agricultural systems, specifically monoculture systems, *via* sustainable intensification or climate-smart agriculture, can reduce the high carbon footprint of agriculture. This can provide an avenue for nature-based transformative adaptation to enable people to adapt to climate change (SDG 13), and mitigate biodiversity loss and reduce land degradation (SDG 15), thereby promoting responsible consumption and production (SDG 12). NUS’s local production can also reduce transportation distances between production and consumption, further reducing agriculture’s carbon footprint (Shelef et al., 2017). The agro-ecological, technological, environmental and socio-economic benefits of NUS and their contribution to the fulfillment of several SDGs, in particular, the goals on food and nutrition security, health and wellbeing, poverty alleviation, climate action, and the preservation of life on land, including the provision of ecosystem services, highlights the importance of these crops in achieving more sustainable, equitable and resilient agricultural and food systems.

### Closing in Toward the Concept of Ecological Integrity Through NUS

**Figure 4:**
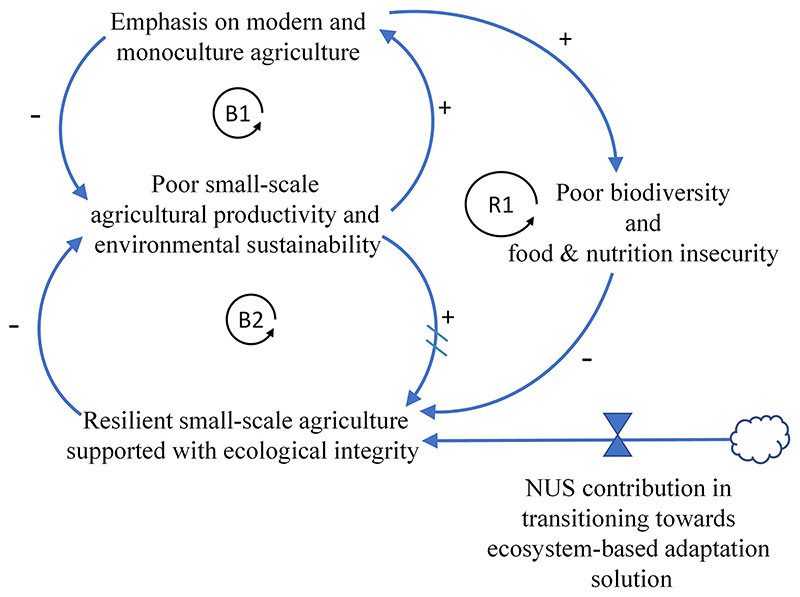
Shifting the burden system archetype. Balancing loop B1 : Illustrates how the conventional policy strategy has sought to address agricultural productivity through monoculture and agrochemical input. R1, a vicious reinforcing loop, shows that modern farming has not always proved sustainable for small-scale agriculture in the long term as biodiversity loss and food and nutrition insecurity have become persistent. Loop R1: Emphasis on modern and monoculture agriculture? Poor biodiversity and food and nutrition insecurity? Resilient small-scale agriculture supported with ecological integrity? Poor small-scale agricultural productivity and environmental sustainability? Emphasis on modern and monoculture agriculture. Loop B2: the long-term vision that seeks to address problems of small-scale farming by developing resilience and ecological integrity. NUS crops are shown to contribute to a transitioning process that encompasses ecosystem-based adaption solutions.

**Figure 5:**
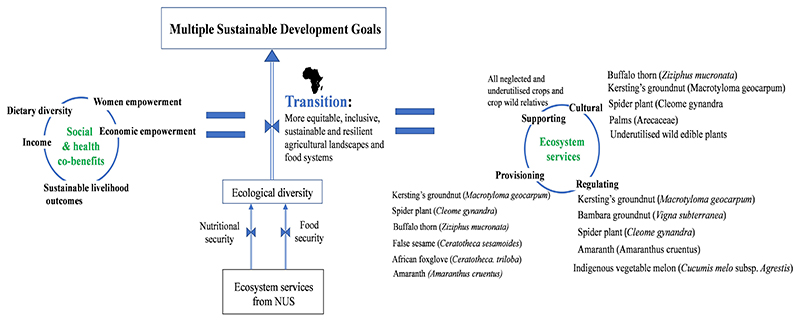
NUS have essentially been considered potential crops to address nutritional and food security. However, from an ecological integrity perspective, NUS could increase on-farm ecological diversity. In effect, the current systematic review has determined the various ecosystem services: supporting, cultural, regulating and provisioning. The focus is on African small-scale agriculture due to the importance of NUS to food and nutrition and culture on the continent (Mayes et al., 2012; Baa-Poku, 2018; Kasolo et al., 2018) and the growing NUS research focus. From an even broader perspective, NUS crops could provide a myriad of social and health co-benefits, including dietary diversity, economic and women empowerment, income and sustainable livelihood outcomes. Altogether, these benefits would form part of a dynamic transition system to achieve multiple Sustainable Development Goals (SDGs).

To the best of the authors’ knowledge, this is the first peer-reviewed contribution that seeks to find common grounds between NUS crops and the contribution of their ecosystem services to ecological integrity on African NUS crops. In effect, the concept of ecological integrity creates a novel perspective on multidisciplinary research on the NUS crops. Since the Green Revolution, policies to achieve food security have focused on yield improvement, emphasizing the use of agrochemical inputs (Dawson et al., 2016). Such an approach has not always proven to be feasible and/or adaptable to the context of small-scale African food production (Leakey, 2018). Food production systems are dynamic and depend on the many infrastructural and institutional pre-conditions to work effectively solely on the assumption that the food security problems can be fixed based on conventional/modern agricultural systems alone (Leakey, 2020). This is why in the present work, the NUS crops are being viewed as a more fundamental solution to resolve small-scale food systems (Figure 4) and are being viewed as one of the many adaptive options that can be adopted to transition (Figure 5) toward resilient and ecologically diverse food systems. The transition is complex and requires transdisciplinary approaches for successful implementation.

The transition will also require overcoming some of the burgeoning challenges within NUS regimes in Africa. These include the low yield of the crops (Mabhaudhi et al., 2017b); the lack of formal markets (Weinberger, 2007; Mabhaudhi et al., 2017b, 2019); the lack of research dedicated to these crops; and the low adoption, especially by the youth, who associate these crops with poverty (Gotor and Irungu, 2010; Rudebjer et al., 2013). As a result, the cultivation of NUS is mainly confined to traditional cropping systems and smallholder farming areas with marginal lands for crop cultivation (Padulosi et al., 2013; Chivenge et al., 2015; Baa-Poku, 2018; Kasolo et al., 2018), and there is little recognition of their potential to contribute toward nutrition security, health, income generation, and environmental services (Jena et al., 2018).

## Policy Implications

The findings of this study are useful for informing and fulfilling the aims of various policies and frameworks related to food, nutrition, health, and biodiversity in Africa, at the regional, sub-regional and national levels (Table 2). Based on the evidence from the review, we developed policy recommendations for key policies and programs within the African Union (AU), Southern African Development Community (SADC), and South Africa, where most of the research on NUS has been conducted (Towns and Shackleton, 2018; Table 2). Mainstreaming NUS into policy and subsequent investment can effectively facilitate transformation and achieve the common goal of sustainable and equitable development.

## Limitations of Study

A possible limitation was restricting search terms to “neglected and underutilized species” and “orphan species.” Several terms are used interchangeably with NUS, even though the terms are not necessarily accurate. Examples of these terms include “edible wild species”, “African leafy vegetables”, “wild foods,” and “traditional food crops,” and “future crop species.” However, the present study took cognisance of these facts and opted to focus the literature search on the term that has become globally acceptable to describe and encompass all these terms—i.e., neglected and underutilized species (Mabhaudhi et al., 2017b). This also aligns the research with emerging global research and increases recognition of research outputs from Africa (Mabhaudhi et al., 2017b).

Furthermore, the search yielded research focusing on priority underutilized crops in Africa and South Africa, which are crops of interest, making the search relevant to current studies. Most articles, however, mainly reported on the positive attributes of NUS, with little reference to their negative attributes, which introduces bias to any research. Moreover, most of the research on NUS is anecdotal and not experimental, making it difficult to support or disprove their supposed benefits. To overcome this bias, the findings were verified with other literature sources.

## Conclusion

In Africa, NUS have historically served as an important source of food and nutrition, and are deeply embedded in different cultures and traditions (Mayes et al., 2012; Baa-Poku, 2018; Kasolo et al., 2018). Through wider use of these crops, diversity and diversification can contribute to more sustainable, equitable and resilient agricultural landscapes and food systems. NUS can help mitigate negative trade-offs related to biodiversity loss, increased susceptibility to pests and plant diseases, land degradation, water contamination and pollution, loss of genetic resources, and greenhouse gas emissions (Shelef et al., 2017; Crews et al., 2018). Modern agriculture is dominated by monoculture farming systems and relies on external inputs such as fertilizer and pesticides; this has had dire impacts on the environment in Africa (Crews et al., 2018). Diversity is an essential factor in improving productivity and enhancing ecosystem functioning, especially in a changing climate. There is a need to change the status quo by transforming agricultural landscapes and food systems, and NUS can be a part of this transformation. However, a trade-off currently exists between increasing the productivity of common crops or investing in crop diversity by utilizing more species (Shelef et al., 2017). A land-use transformation of this kind requires more financial and time investments; however, it will achieve long-term results by making agricultural production systems more resilient and sustainable while reducing the vulnerability of the population, increasing its adaptive capacity, and promoting self-reliance.

NUS not only have agro-ecological, technological, environmental and socio-economic elements and benefits. They can also serve to improve quality of life by providing dietary diversity and household food and nutrition security, health benefits, livelihood and income for the poor, and empowering women economically, contributing to gender equity. Thus, they can serve as a tool for transformative ecosystem-based adaptation to climate change. Moreover, the potential for NUS to achieve sustainable livelihood outcomes illustrates their potential to address the root causes of vulnerability (Fedele et al., 2019) while providing services for human wellbeing and shifting away from unsustainable practices. We propose that NUS be embedded into strategies to develop climate-resilient diets and food systems; furthermore, the role of women in the production systems of NUS need to be acknowledged as an important additional benefit.

Future research should take an in-depth analysis of the potential of NUS to build livelihood capital. This could build a stronger case for the adoption of NUS by providing a livelihoods perspective and food and nutrition, and environmental perspective. Education and training on NUS, as well as market access, will be needed in order the improve livelihoods. Another study can also be conducted on the gendered perspective of NUS to ensure equitable livelihood improvement and improved quality of life.

## Supplementary Material

Supplemental Material

## Figures and Tables

**Table 1:** The contribution of selected NUS in Africa to the four ecosystem service areas.

NUS	Geographical area	Specific benefit	Implications in terms of sustainability	References
**Provisioning**				
Kersting’s groundnut *(Macrotyloma geocarpum)*	West Africa-Benin, Ghana, Northern Togo Nigeria	*Food and nutrition*-cheapest source of protein for rural populations *Medicinal*-reduces the occurrence of atherosclerosis and coronary heart disease (as it has a low fat [1.0 %) and sodium content (5.67 mg g^−1^)] The groundnut is also a good source of iron which makes it good for patients with anemia	Food system transformation Social and economic sustainability	Ayenan and Ezin, 2016
Spider plant *(Cleome gynandra)*	Botswana Kenya Malawi Tanzania Uganda Zambia Zimbabwe	*Food and nutrition-* the tender leaves or young shoots, and often the flowers are eaten boiled as a pot herb, tasty relish, stew, or side dish, as an accompaniment with cereal-based foods *Medicinal*-rich in iron and good for patients recuperating from surgery or serious illness; the vegetable is good for expectant mothers to help them deliver easily and to stimulate milk let-down and production	Social and economic sustainability	Onyango et al., 2013
Buffalo thorn *(Ziziphus mucronata)*	Angola Benin, Botswana Cameroon, Eritrea Ethiopia, Kenya Mali Namibia Niger Nigeria Senegal South Africa Tanzania Zambia Zimbabwe	*Medicinal*-traditional medicinal treatment of arthritis, muscle pains, chest pains, headache, colds, measles, swollen organs, and liver problems *Food and nutrition*-the leaves are edible and young ones can be cooked and eaten like spinach. Sometimes the fruits are sucked by children and are reportedly sold in rural markets in Zimbabwe *Feed and fodder*-a valuable source of food for all browsers such as giraffes, springboks, antelopes, black rhinos, and elephants Its highly nutritious fruits are usually eaten by monkeys, baboons, warthog, and birds They play a central role in the nutrition of larval caterpillars, such as *Tuxentius melaena, T. calice*, and *Zintha hintza*	Social and economic sustainability	Mokgolodi et al., 2011
False sesame (*Ceratotheca sesamoides)*	Nigeria South Africa Mali Burkina Faso	*Food and nutrition*-High in macronutrients *Medicinal*-has anti-diarrhoeal, anti-diabetic, antiplasmodial, antibacterial, and antiviral properties In Nigeria, *C. sesamoides* is used in the treatment of diarrhea, conjunctivitis, snakebites, skin diseases and facilitation of birth in both humans and animals	Social sustainability	Masondo et al., 2016
African foxglove (*Ceratotheca. triloba*)	Nigeria South Africa	*Food and nutrition-* High in macronutrients-cooked as spinach *Medicinal* -High in macronutrients and has anti-diarrhoeal, anti-diabetic, antiplasmodial, antibacterial, and antiviral properties In South Africa, *C. triloba* leaves are extensively utilized by Zulu people for the relief of painful menstruation, stomach cramps and diarrhea	Social and economic sustainability	Masondo et al., 2016
Amaranth *(Amaranthus cruentus)*	Kenya Nigeria Sudan Tanzania	*Food and nutrition-* leaves contain high levels of β-carotene	Social and economic sustainability	Hughes and Ebert, 2013
**Regulating**				
Kersting’s groundnut (*Macrotyloma geocarpum*)	West Africa-Benin Ghana, Northern Togo Nigeria	*Erosion prevention and soil fertility*-enhance soil fertility by fixing nitrogen (N2) in drought-prone environments where other crops may not survive *Biological control*-Kersting’s groundnut can fix up to 16.5-57.8kg ha^−1^ of atmospheric N2	Environmental sustainability	Tele Ayenan and Ezin, 2016
Bambara groundnut (*Vigna subterranea*)	Niger North Africa Southern Africa	*Erosion prevention and soil fertility*-enhance soil fertility by fixing nitrogen (N2) in drought-prone environments where other crops may not survive	Environmental sustainability	Chivenge et al., 2015
Spider plant (*Cleome gynandra*)	Kenya	*Biological control-* C4 plants can tolerate water and temperature stress and thrive in hot and arid environments. This is due to the carbon fixation mode of C4 plants, which enables them to photosynthesise more efficiently, and experience reduced water loss by transpiration. Resistant to whiteflies, beetles, leaf-miners and brown coreid bugs	Environmental sustainability-the carbon fixation mode of C4 plants enables them to photosynthesise more efficiently, and experience reduced water loss by transpiration, increasing their resilience. Advantageous in the face of climate change and uncertainty, where changes in temperature and rainfall patterns affect crop production and alter the incidence and distribution of insect pests and diseases. This increases the biotic stress on crop production systems	Hughes and Ebert, 2013
Amaranth *(Amaranthus cruentus)*	Kenya Nigeria Sudan Tanzania	*Biological control-* C4 plants can tolerate water and temperature stress and thrive in hot and arid environments. This is due to the carbon fixation mode of C4 plants, which enables them to photosynthesise more efficiently, and experience reduced water loss by transpiration. Resistant to root-knot nematodes	Environmental sustainability is less susceptible to pests and diseases and, therefore, requires fewer pesticide and fertilizer interventions. Low requirement and use of agrochemicals can mitigate the negative impacts that agrochemicals have on the environment and promote biodiversity and maintain ecosystem health	Hughes and Ebert, 2013
Indigenous vegetable melon (*Cucumis melo subsp. Agrestis*)	Sudan	Resistant to several pests, including leaf miners, fruit flies, and diseases such as Fusarium wilt (*Fusarium oxysporum*), powdery mildew, *Zucchini yellow mosaic virus* (*Potyvirus*), *Cucurbit aphid-borne yellows virus* (*Polerovirus*)	Environmental sustainability due to resistance to pests and diseases and low requirement and use of agrochemicals	Hughes and Ebert, 2013
**Habitat and support**				
All neglected and underutilized crops and crop wild relatives	Africa	*Genetic diversity*	Environmental/ecological sustainability	Quartermain, 2007; Gotor and Irungu, 2010; Hughes and Ebert, 2013; Rudebjer et al., 2013; Nyadanu and Lowor, 2014; Chivenge et al., 2015; Karunaratne et al., 2015; Masondo et al., 2016; Nyadanu et al., 2016; Tele Ayenan and Ezin, 2016; Mabhaudhi et al., 2017a,b; Towns and Shackleton, 2018; Campanaro et al., 2019
**Cultural**				
Buffalo thorn *(Ziziphus mucronata*)	Botswana South Africa Swaziland	Used in connection with burial rites by the Zulu and Swazi tribes. Believed to be immune to lightning. Anyone sheltering under the crop in a storm is considered to be safe	Social sustainability	Mokgolodi et al., 2011
Kersting’s groundnut *(Macrotyloma geocarpum)*	Northern Ghana Togo	Dried boiled seed of Kersting’s groundnut has cultural and symbolic value for the Sisala people. The seeds are served to children during the final funeral rites of their mother. In Togo, the seeds are used by the Kabyes and Maubas people in rituals and funeral ceremonies	Social sustainability	Tele Ayenan and Ezin, 2016
Spider plant *(Cleome gynandra)*	Nigeria	Spider plant leaves are boiled, butter added, and eaten with ugali made from finger millet flour, and is served to important visitors such as in-laws as a sign of respect	Social sustainability	Onyango et al., 2013
Palms *(Arecaceae)*	Madagascar	Spiritual and cultural values and are often used for spiritual healing	Social sustainability	Gruca et al., 2016
Underutilized wild edible plants	Ethiopia Botswana South Africa	Recreational value	Social sustainability	Tebkew et al., 2014

**Table 2:** Policy implications of NUS research and contribution to the fulfillment of global, African, regional (southern Africa), and national (South Africa) goals.

Name of policy	Description of policy	NUS ES relevant to the policy
Global policies		
World Declaration and Plan of Action for Nutrition (1992) FAO WHO, 1992	It aims to ensure all people have continued access to sufficient supplies of safe foods for a nutritionally adequate diet; achieve and maintain health and nutritional wellbeing of all people; and achieve developmental goals that are sustainable, environmentally sound and contribute to improved nutrition and health.	The introduction of NUS into the global food diet can diversify agricultural production systems safely while providing healthy and nutritious food.
Rome Declaration on World Food Security (1996) (FAO, 1996)	Commitment to: ensuring an enabling political, social, and economic environment designed to create the best conditions for the eradication of poverty and durable peace, based on full and equal participation of women and men, which is most conducive to achieving sustainable food security for all; implementing policies aimed at eradicating poverty and inequality and improving physical and economic access by all, always, to sufficient, nutritionally adequate, and safe food and its effective utilization; the pursuit of participatory and sustainable food, agriculture, fisheries, forestry and rural development policies and practices in high and low potential areas, which are essential to adequate and reliable food supplies at the household, national, regional and global levels, and combat pests, drought and desertification, considering the multifunctional character of agriculture; ensuring food, agricultural trade, and overall trade policies are conducive to fostering food security for all through a fair and market-oriented world trade system; preventing and being prepared for natural disasters and man-made emergencies and to meet transitory and emergency food requirements in ways that encourage recovery, rehabilitation, development, and a capacity to satisfy future needs; promoting optimal allocation and use of public and private investments to foster human resources, sustainable food, agriculture, fisheries and forestry systems, and rural development, in high and low potential areas; and implementing, monitor, and follow-up this Plan of Action at all levels in cooperation with the international community.	Strategies focusing on NUS can assist with meeting some of these goals, including sustainable food and nutrition security and eradicating poverty and inequality.
**African policies**		
The 2014 Malabo Declaration on Accelerated Agricultural Growth and Transformation for Shared Prosperity and Improved Livelihoods (AU, 2016)	Commitment to end hunger in Africa by 2025. These commitments can be achieved through inclusive agricultural growth and transformation, a commitment to enhancing the resilience of livelihoods and production systems to climate variability and other related risks.	The transformation can be facilitated by introducing NUS into agricultural production systems, which can improve the resilience of both the food system and livelihoods while promoting inclusivity and gender equality.
The Comprehensive Africa Agriculture Development Programme (CAADP) of New Partnership for Africa’s Development (NEPAD) (NEPAD, 2019)	The focus of this policy is to improve agricultural productivity and increase public investment in agriculture.	The introduction of NUS can diversify agricultural production systems, with fewer inputs required, enriching ecosystems, and making the cropping systems more sustainable, ultimately improving nutrition and food security on the continent.
The 2013 Southern African Development Community (SADC) Regional Agricultural Policy (SADC, 2013)	It aims to contribute to sustainable agricultural growth and socio-economic development. This is achieved through various strategies, including, among others, increased productivity, and competitiveness, improving regional and international trade, and access to markets for agricultural products.	A strategy focussing on underutilized resources such as NUS can assist with meeting some of these goals.
**South African policies and legislation**		
Constitution of South Africa (1996) (Republic of South Africa, 1996)	The Bill of Rights provides the basic human rights of South Africans, including the rights to dignity, the right to have the environment protected for sustainable use and social and economic development, sufficient food to participate in the cultural life of their choice.	Including traditional crops in agricultural systems, enhanced by traditional knowledge and practices, respects the rights and culture of indigenous people, thereby providing dignity. Environmental, social, and economic benefits of NUS promote sustainable development, improve livelihoods, and reduce food insecurity.
The White Paper of National Climate Change Response (DEA, 2011)	It aims to transition the country into a climate-resilient, equitable and internationally competitive lower-carbon economy and society while simultaneously addressing sustainable development, job creation, improved public and environmental health, poverty eradication, and social equality.	The integration of NUS can provide environmental and health co-benefits, improve livelihoods, reduce carbon footprints, and promote gender equality.
The 2012 National Development Plan (NDP) (National Planning Commission, 2012)	It aims to eliminate poverty and reduce inequality by 2030.	The incorporation of NUS can provide marginalized South Africans with a means to earn an income to ensure household food and nutrition security, independent of government intervention.
National Policy on Plant Improvement (DAFF, 2013)	It aims to provide a broad framework for supporting and regulating the production and trade of propagation material and related matters to support the government’s commitment to food security, job creation, and economic development.	Given the genetically diverse nature of NUS, the policy would benefit from supporting research and production of NUS to contribute to food security and job creation in the country. Likewise, incorporating NUS into this policy could facilitate the integration of NUS into the food diet.
The Strategic Plan for the Prevention and Control of Non-Communicable Diseases 2013-17 (Department of Health, 2013)	It aims to decrease the incidence of NCDs and achieve long and healthy lives for all.	This study showed that NUS’s nutritional and chemical diversity could potentially enable them to reduce the incidence of non-communicable diseases (NCDs), which is particularly relevant in South Africa, where there is a high incidence of NCDs.
The National Policy on Food and Nutrition Security (DAFF, 2014)	Seeks ensure the availability, accessibility, and affordability of safe and nutritious food at national and household levels.	The integration of NUS into any strategies aimed at achieving this goal would be beneficial, as it is locally available, nutritious, and the cheapest source of macro- and micro-nutrients
The National Climate Change and Health Adaptation Plan (2014-2019) (Department of Health, 2014)	It aims to provide a broad framework for health sector action toward adaptation to climate change.	Incorporating NUS into this plan can help the country sustain socio-economic and environmental resilience through its various environmental and health benefits.
The Republic of South Africa Department of Health Strategic Plan 2015/16 - 2019/20 (Department of Health, 2015)	It aims to improve health status by preventing illness, disease and promoting healthy lifestyles while strengthening the health care delivery system.	NUS have numerous health and medicinal properties and are more affordable than pharmaceutical medications. Mainstreaming NUS into this plan may help to improve health care for all in the country, especially the poor.
The National Biodiversity Economy Strategy (2016) (DEA, 2016))	Seeks to optimize the economic benefits from the sustainable use of South Africa’s biodiversity.	NUS could contribute to rural communities’ income and livelihood strategies as most of the indigenous biological resources surround these communities; thereby, assisting with the country’s transition to a green economy through job creation and poverty reduction. This Strategy will also play a major role in the transformation of the economy by motivating marginalized individuals to start their biodiversity-based enterprises and enhancing the entrepreneurial spirit of current players in the sector, thus creating a palpable and sustainable economic presence. The commercial or industrial utilization of the indigenous biological/ genetic resources such as NUS in biodiversity economy sectors offers the opportunity to create additional employment. The effective implementation of the legislative provisions (namely, National Environmental Management Biodiversity Act No. 10 of 2004) on the use of indigenous biological/ genetic resources and the effective support of small enterprises in this field rely on sound knowledge and understanding of the biodiversity economy.
The National Climate Change Adaptation Strategy (DEA, 2019)	Seeks to transition the country to climate resilience while fulfilling its development aspirations.	Policy makers and practitioners must consider implementing ecosystem-based transformative adaptation that incorporates NUS.

## Data Availability

The original contributions presented in the study are included in the article/**Supplementary Material**, further inquiries can be directed to the corresponding author.
