# Supplementary material for: Diversity and Diversification: Ecosystem Services Derived From Underutilized Crops and Their Co-benefits for Sustainable Agricultural Landscapes and Resilient Food Systems in Africa
Source: Front Agron. Author manuscript; Available in PMC 2023 Sep 7. (PMC7615041; doi:10.3389/fagro.2022.859223)
Supplement: Supplemental Material [file EMS187256-supplement-Supplemental_Material.DOCX]

Supplementary Material

1. **Supplementary Information 1**

## The Economics of Ecosystems and Biodiversity (TEEB)

The Economics of Ecosystems and Biodiversity (TEEB) is a global initiative which was launched in 2007 by Germany and the [European Commission](https://en.wikipedia.org/wiki/European_Commission) with the goals of valuating nature by analysing (i) the global economic benefit of biological diversity, (ii) the costs of the loss of biodiversity, and (iii) the failure to take protective measures versus the costs of effective conservation (TEEB, 2010). Therefore, the aim of TEEB is to mainstream biodiversity and ecosystem services in all levels of decision-making by forming interlinkages between the multi-disciplinary science of biodiversity, policy, and government and business practices (TEEB, 2010).

In order to undertake the global study, TEEB took inspiration from ideas developed by the Millennium Ecosystem Assessment (MEA) (TEEB, 2008). The initiative modified the ecosystem categories that were provided by the MEA to address the link between biodiversity and ecosystem services (Evers et al., 2018). Another major change was the removal of supporting services from the MEA as it would result in double-counting in economic assessments, and because it is more of an ecosystem function than a service (Pérez-Soba et al., 2012). The supporting category was subsequently replaced by habitat and support (TEEB, 2008).The current study used the updated TEEB categories as a guide to determine the types of ecosystem services provided by NUS. However, we also modified these categories to focus on the ecosystem services that are linked to the food system as a whole, and excluded the services that we found not to have a direct link to the food system and NUS (see Table 1 in Supplementary Information 2). Therefore, the four types of ecosystem services investigated were provisioning, regulating, habitat and support, and cultural services***.***

1. **Supplementary Information 2**

Table 2: Classification of ecosystem services potentially provided by NUS, modified from TEEB

| **PROVISIONING** | **REGULATING** | **HABITAT & SUPPORT** | **CULTURAL** |
| --- | --- | --- | --- |
| Food | Local climate and air quality- Air purifying, shade | Habitat for species | Eco-tourism, cultural, tourism |
| Raw materials- wood, biofuels | Carbon sequestration and storage- C sequestration, C storage | Maintenance of genetic diversity | Aesthetic, cultural heritage and knowledge and education |
| Freshwater- hydrological cycle, regulate flow and purifies | Moderation of extreme events- e.g. Tree stabilize slopes |  | Spiritual and Religious, sense of place |
| Medicinal- Traditional medicine; pharmaceutical industry | Erosion prevention and soil fertility- erosion control, soil nutrients |  |  |
|  | Pollination |  |  |
|  | Biological control- Bio-control, pest control |  |  |

1. **Supplementary Information 3**

**Table 3**: Search Terms

| **PROVISIONING** |  |
| --- | --- |
| 1. Food | Food, nutrition, productivity, harvest, yield, biomass |
| 1. Raw materials | Raw material, wood, fibre, building material, timber, forest resources, fuel wood, fire wood, biofuel, biomass |
| 1. Fresh water | Flow regulation, water quality, water quantity, hydrological cycle, water purification, water flow, water regulation, freshwater, blue water, water regulation, surface water |
| 1. Medicinal | Medicinal, traditional medicine, muthi*, curative, pharmaceutical |
| **REGULATING** |  |
| 1. Local climate and air quality | Air purification, shade, air quality, local climate, microclimate, cooling |
| 1. Carbon sequestration and Storage | Carbon, carbon storage, carbon sequestration, carbon stock, carbon cycle, soil carbon, biomass |
| 1. Moderation of extreme events | Buffer, biodiversity, multi-cropping, hedgerows, trees, flood, storm, land slide, extreme weather, extreme events, drought tolerance, deep roots, mud slide |
| 1. Erosion prevention and soil fertility | Erosion prevention, soil fertility, erosion control, soil nutrients, buffer, biodiversity, multi-cropping, hedgerows, trees, land slide, extreme events |
| 1. Pollination | Pollinators, pollination |
| 1. Biological control | Biological control, Pest control, bio efficacy, pest regulation, deterrents, pubescent |
| **HABITAT AND SUPPORT** |  |
| 1. Habitat of species | Habitat, invertebrates, rodents, biodiversity, landscape diversity/heterogeneity, heterogeneous |
| 1. Maintenance of genetic diversity | Genetic diversity, ecological diversity, genetic storage, agroecological diversity, gene pool |
| **CULTURAL** |  |
| 1. Eco- and cultural tourism | Eco-tourism, cultural tourism, cultural village |
| 1. Aesthetic, cultural heritage and knowledge and education | Aesthetic, cultural heritage, knowledge, education, beauty, scenic, peace, creation, indigenous knowledge systems, tradition, ceremony biodiversity, inspiration |
| 1. Spiritual and Religious, sense of place | Spiritual, religious, ancestors, prayer, worship, sense of place, belonging, sovereignty, customs, traditional knowledge |

*** local language**

**METHODS FOR LITERATURE SEARCH**

Within SCOPUS, search terms were set to be in the title, abstract and keywords. The search terms included: “underutilized” or “neglected crops” in relation to “ecosystem services”, “provisioning services” (food, raw materials, freshwater, medicinal properties), “regulating services” (local climate and air quality, carbon sequestration and storage, moderation of extreme events, erosion prevention and soil fertility, pollination, biological control), “habitat and support” (genetic diversity) , and “cultural services” (eco- and cultural tourism, aesthetic, cultural heritage, knowledge and education, spiritual and religious sense of place) (see Table 3 in Supplementary Information 3). In cases where each search term retrieved less than 100 hits, the search string was separated into smaller components, containing two key terms.

**Search Strings**

( TITLE-ABS-KEY ( neglected  AND  underutilised  AND crops )  AND  TITLE-ABS-KEY ( ecosystem  AND services ) ) (**102 DOCUMENTS)**

**( TITLE-ABS-KEY ( neglected  AND  underutilised  AND crops )  AND  TITLE-ABS-KEY ( ecosystem  AND services ) ) (50 DOCUMENTS)**

TITLE-ABS-KEY ( ( "NEGLECTED AND UNDERUTILISED"  OR  "NEGLECTED"  OR  "UNDERUTILISED"  OR  "ORPHAN" )  AND  ( "CROP*"  OR  "SPECIES"  OR  "TREE*" )  AND  ( "ECOSYSTEM SERVICE*" ) )  (**2 DOCUMENTS)**

[TITLE-ABS-KEY ( ( ( "neglected and underutilized"  OR  "NEGLECTED"  OR  "underutilised"  OR "ORPHAN" )  AND  ( "CROP*"  OR  "SPECIES"  OR  "TREE*" )  AND  ( "ECOSYSTEM SERVICE*" ) ) )  AND NOT vertebrate*  OR  animal*](https://www.scopus.com/results/documentSpellSuggest.uri?sort=plf-f&src=s&sid=abd6bb1046cbe0b7ed749ab11c94bf44&sot=a&sdt=a&sl=201&s=TITLE-ABS-KEY+%28+%28+%28+%22neglected+and+underutilized%22+OR+%22NEGLECTED%22+OR+%22underutilized%22+OR+%22ORPHAN%22+%29+AND+%28+%22CROP*%22+OR+%22SPECIES%22+OR+%22TREE*%22+%29+AND+%28+%22ECOSYSTEM+SERVICE*%22+%29+%29+%29+AND+NOT+VERTEBRATE*+OR+ANIMAL*&origin=resultslist) (**0 DOCUMENTS)**

1. **PROVISIONING SERVICES**

TITLE-ABS-KEY ( ( "NEGLECTED AND UNDERUTILISED"  OR  "NEGLECTED"  OR  "UNDERUTILISED"  OR  "ORPHAN" )  AND  ( "CROP*"  OR  "SPECIES"  OR  "TREE*" )  AND  ( "PROVISIONING SERVICE*" ) ) **4 DOCUMENTS)**

**Food:**

TITLE-ABS-KEY ( ( "NEGLECTED AND UNDERUTILISED"  OR  "NEGLECTED"  OR  "UNDERUTILISED"  OR  "ORPHAN" )  AND  ( "CROP*"  OR  "SPECIES"  OR  "TREE*" )  AND  ("FOOD” OR “nutrition” OR “productivity” OR “harvest*” OR “yield” OR “biomass " ) )  **(2612 DOCUMENTS)**

TITLE-ABS-KEY ( ( "NEGLECTED AND UNDERUTILISED"  OR  "NEGLECTED"  OR  "UNDERUTILISED"  OR  "ORPHAN" )  AND  ( "CROP*"  OR  "SPECIES"  OR  "TREE*" )  AND  ( "FOOD"  OR  "nutrition"  OR  "productivity"  OR  "harvest*"  OR  "yield"  OR  "biomass " ) )  AND NOT  vertebrate*  OR  animal*  **(1647 DOCUMENTS)**

**Raw materials:**

TITLE-ABS-KEY ( ( "NEGLECTED AND UNDERUTILISED"  OR  "NEGLECTED"  OR  "UNDERUTILISED"  OR  "ORPHAN" )  AND  ( "CROP*"  OR  "SPECIES"  OR  "TREE*" )  AND  ( " Raw material*” OR “wood” OR “fibre” OR “building material*” OR “timber” OR “forest resource*” OR “fuel wood” OR “fire wood” OR “biofuel*” OR “biomass " ) ) **(960 DOCUMENTS)**

TITLE-ABS-KEY ( ( "NEGLECTED AND UNDERUTILISED"  OR  "NEGLECTED"  OR  "UNDERUTILISED"  OR  "ORPHAN" )  AND  ( "CROP*"  OR  "SPECIES"  OR  "TREE*" )  AND  ( " Raw material*” OR “wood” OR “fibre” OR “building material*” OR “timber” OR “forest resource*” OR “fuel wood” OR “fire wood” OR “biofuel*” OR “biomass " ) )  AND NOT vertebrate*  OR  animal* **(673 DOCUMENTS)**

**Fresh water:**

TITLE-ABS-KEY ( ( "NEGLECTED AND UNDERUTILISED"  OR  "NEGLECTED"  OR  "UNDERUTILISED"  OR  "ORPHAN" )  AND  ( "CROP*"  OR  "SPECIES"  OR  "TREE*" )  AND  ( " Flow regulation” OR “water quality” OR “water quantity” OR “hydrological cycle” OR “water purification” OR “water flow” OR “water regulation” OR “freshwater” OR “blue water” OR “water regulation” OR “surface water" ) ) **(24 DOCUMENTS)**

TITLE-ABS-KEY ( ( "neglected and underutilized"  OR  "NEGLECTED"  OR  "underutilized"  OR  "ORPHAN" )  AND  ( "CROP*"  OR  "SPECIES"  OR  "TREE*" )  AND  ( " Flow regulation"  OR  "water quality"  OR  "water quantity"  OR  "hydrological cycle"  OR  "water purification"  ,  "water flow"  OR  "water regulation"  OR  "freshwater"  OR  "blue water"  OR  "water regulation"  OR  "surface water" ) )  AND NOT  vertebrate*  OR  animal* **(16 DOCUMENTS)**

**Medicinal:**

TITLE-ABS-KEY ( ( "NEGLECTED AND UNDERUTILISED"  OR  "NEGLECTED"  OR  "UNDERUTILISED"  OR  "ORPHAN" )  AND  ( "CROP*"  OR  "SPECIES"  OR  "TREE*" )  AND  ( "Medicin*” OR “traditional medicine” OR “muthi*” OR “curative” OR “pharmaceutical" ) ) **(711 DOCUMENTS)**

TITLE-ABS-KEY ( ( "NEGLECTED AND UNDERUTILISED"  OR  "NEGLECTED"  OR  "UNDERUTILISED"  OR  "ORPHAN" )  AND  ( "CROP*"  OR  "SPECIES"  OR  "TREE*" )  AND  ( "Medicin*"  OR  "traditional medicine"  ,  "muthi*"  OR  "curative"  OR  "pharmaceutical" ) )  AND NOT  vertebrate*  OR  animal* **(24 DOCUMENTS)**

1. **REGULATING SERVICES**

TITLE-ABS-KEY ( ( "NEGLECTED AND UNDERUTILISED"  OR  "NEGLECTED"  OR  "UNDERUTILISED"  OR  "ORPHAN" )  AND  ( "CROP*"  OR  "SPECIES"  OR  "TREE*" )  AND  ( "REGULATING SERVICE*" ) ) **( 4 DOCUMENTS)**

**Local climate and air quality:**

TITLE-ABS-KEY ( ( "NEGLECTED AND UNDERUTILISED"  OR  "NEGLECTED"  OR  "UNDERUTILISED"  OR  "ORPHAN" )  AND  ( "CROP*"  OR  "SPECIES"  OR  "TREE*" )  AND  ( "Air purif*” OR “shade” OR “air quality” OR “local climate” OR “microclimate” OR “cooling”)) **(118 DOCUMENTS)**

TITLE-ABS-KEY ( ( "NEGLECTED AND UNDERUTILISED"  OR  "NEGLECTED"  OR  "UNDERUTILISED"  OR  "ORPHAN" )  AND  ( "CROP*"  OR  "SPECIES"  OR  "TREE*" )  AND  ( "Air purif*” OR “shade” OR “air quality” OR “local climate” OR “microclimate” OR “cooling”)) AND NOT  vertebrate*  OR  animal* **(102 DOCUMENTS)**

**Carbon sequestration and Storage:**

TITLE-ABS-KEY ( ( “NEGLECTED AND UNDERUTILISED”  OR  “NEGLECTED”  OR  “UNDERUTILISED”  OR  “ORPHAN” )  AND  ( “CROP*”  OR  “SPECIES”  OR  “TREE*” )  AND  ( “ Carbon” OR “carbon storage” OR “carbon sequestration” OR “carbon stock” OR “carbon cycle” OR “soil carbon” OR “biomass” ) ) **(791 DOCUMENTS)**

TITLE-ABS-KEY ( ( “NEGLECTED AND UNDERUTILISED”  OR  “NEGLECTED”  OR  “UNDERUTILISED”  OR  “ORPHAN” )  AND  ( “CROP*”  OR  “SPECIES”  OR  “TREE*” )  AND  ( “ Carbon”  OR  “carbon storage”  OR  “carbon sequestration”  OR  “carbon stock”  OR  “carbon cycle”  OR  "soil carbon"  OR  "biomass" ) )  AND NOT  vertebrate*  OR  animal*  **(568 DOCUMENTS)**

**Moderation of extreme events:**

TITLE-ABS-KEY ( ( "NEGLECTED AND UNDERUTILISED"  OR  "NEGLECTED"  OR  "UNDERUTILISED"  OR  "ORPHAN" )  AND  ( "CROP*"  OR  "SPECIES"  OR  "TREE*" )  AND  (" Buffer*” OR “biodiversity” OR “multi-cropping” OR “hedgerow*” OR “tree*” OR “flood*” OR “storm*” OR “land slide*” OR “extreme weather” OR “extreme events” OR “drought tolerance” OR “deep root*” OR “mud slide*” ) ) **(2798 DOCUMENTS)**

TITLE-ABS-KEY ( ( "NEGLECTED AND UNDERUTILISED"  OR  "NEGLECTED"  OR  "UNDERUTILISED"  OR  "ORPHAN" )  AND  ( "CROP*"  OR  "SPECIES"  OR  "TREE*" )  AND  ( " Buffer*"  OR  "biodiversity"  OR  "multi-cropping"  OR  "hedgerow*"  OR  "tree*"  OR  "flood*"  OR  "storm*"  OR  "land slide*"  OR  "extreme weather"  OR  "extreme events"  OR  "drought tolerance"  OR  "deep root*"  OR  "mud slide*" ) )  AND NOT  vertebrate*  OR  animal* **(1870 DOCUMENTS)**

**Erosion prevention and soil fertility:**

TITLE-ABS-KEY ( ( "NEGLECTED AND UNDERUTILISED"  OR  "NEGLECTED"  OR  "UNDERUTILISED"  OR  "ORPHAN" )  AND  ( "CROP*"  OR  "SPECIES"  OR  "TREE*" )  AND  ( "Erosion prevention” OR “soil fertility” OR “erosion control” OR “soil nutrient” OR “buffer” OR “biodiversity” OR “multi-cropping” OR “hedgerow*” OR tree* OR “land slide” OR “extreme event*”) ) **(2720 DOCUMENTS)**

TITLE-ABS-KEY ( ( "NEGLECTED AND UNDERUTILISED"  OR  "NEGLECTED"  OR  "UNDERUTILISED"  OR  "ORPHAN" )  AND  ( "CROP*"  OR  "SPECIES"  OR  "TREE*" )  AND  ( "Erosion prevention"  OR  "soil fertility"  OR  "erosion control"  OR  "soil nutrient"  OR  "buffer"  OR  "biodiversity"  OR  "multi-cropping"  OR  "hedgerow*"  OR  tree*  OR  "land slide"  OR  "extreme event*" ) )  AND NOT  vertebrate*  OR  animal* **(1818 DOCUMENTS)**

**Pollination:**

TITLE-ABS-KEY ( ( "NEGLECTED AND UNDERUTILISED"  OR  "NEGLECTED"  OR  "UNDERUTILISED"  OR  "ORPHAN" )  AND  ( "CROP*"  OR  "SPECIES"  OR  "TREE*" )  AND  ( “Pollinate” OR " Pollinator*” OR “pollination *" ) ) **(114 DOCUMENTS)**

TITLE-ABS-KEY ( ( "NEGLECTED AND UNDERUTILISED"  OR  "NEGLECTED"  OR  "UNDERUTILISED"  OR  "ORPHAN" )  AND  ( "CROP*"  OR  "SPECIES"  OR  "TREE*" )  AND  ( " Pollinate” OR “Pollinator*” OR “pollination " ) )  AND NOT  vertebrate*  OR  animal* **(42 DOCUMENTS)**

**Biological control:**

TITLE-ABS-KEY ( ( “NEGLECTED AND UNDERUTILISED”  OR  “NEGLECTED”  OR  “UNDERUTILISED”  OR  “ORPHAN” )  AND  ( “CROP*”  OR  “SPECIES”  OR  “TREE*” )  AND  ( “Biological control” OR “Pest control” OR “bio efficacy” OR “pest regulation” OR “deterrent*” OR “pubescent*” ) ) **(119 DOCUMENTS)**

TITLE-ABS-KEY ( ( “NEGLECTED AND UNDERUTILISED”  OR  “NEGLECTED”  OR  “UNDERUTILISED”  OR  “ORPHAN” )  AND  ( “CROP*”  OR  “SPECIES”  OR  “TREE*” )  AND  ( “Biological control” OR “Pest control” OR “bio efficacy” OR “pest regulation” OR “deterrent*” OR “pubescent*” ) ) AND NOT  vertebrate*  OR  animal* **(52 DOCUMENTS)**

1. **HABITAT AND SUPPORT**

TITLE-ABS-KEY ( ( “NEGLECTED AND UNDERUTILISED”  OR  “NEGLECTED”  OR  “UNDERUTILISED”  OR  “ORPHAN” )  AND  (“CROP*”  OR  “SPECIES”  OR  “TREE*” )  AND  ( “ HABITAT AND SUPPORT” ) )  **(0 DOCUMENTS)**

TITLE-ABS-KEY ( ( “NEGLECTED AND UNDERUTILISED”  OR  “NEGLECTED”  OR  “UNDERUTILISED”  OR  “ORPHAN” )  AND  (“CROP*”  OR  “SPECIES”  OR  “TREE*” )  AND  ( “ SUPPORTING SERVICE” ) )**(4 DOCUMENTS)**

TITLE-ABS-KEY ( ( “NEGLECTED AND UNDERUTILISED”  OR  “NEGLECTED”  OR  “UNDERUTILISED”  OR  “ORPHAN” )  AND  (“CROP*”  OR  “SPECIES”  OR  “TREE*” )  AND  ( “ HABITAT” ) )**(880 DOCUMENTS)**

TITLE-ABS-KEY ( (“NEGLECTED AND UNDERUTILISED”  OR  “NEGLECTED”  OR  “UNDERUTILISED”  OR  “ORPHAN” )  AND  ( “CROP*”  OR  “SPECIES”  OR  “TREE*” )  AND  ( “ HABITAT AND SUPPORT” ) ) AND NOT  vertebrate*  OR  animal* **(0 DOCUMENTS)**

TITLE-ABS-KEY ( ( "neglected and underutilized"  OR  "NEGLECTED"  OR  "underutilized"  OR  "ORPHAN" )  AND  ( "CROP*"  OR  "SPECIES"  OR  "TREE*" )  AND  ( "supporting service" )  AND NOT  vertebrate*  OR  animal* ) **(2 DOCUMENTS)**

TITLE-ABS-KEY ( ( "neglected and underutilized"  OR  "NEGLECTED"  OR  "underutilized"  OR  "ORPHAN" )  AND  ( "CROP*"  OR  "SPECIES"  OR  "TREE*" )  AND  ( "habitat" )  AND NOT  vertebrate*  OR  animal* ) **(581 DOCUMENTS)**

**Habitat of species:**

TITLE-ABS-KEY ( ( "NEGLECTED AND UNDERUTILISED"  OR  "NEGLECTED"  OR  "UNDERUTILISED"  OR  "ORPHAN" )  AND  ( "CROP*"  OR  "SPECIES"  OR  "TREE*" )  AND  ("Habitat” OR “invertebrates” OR “rodents” OR “biodiversity” OR “landscape diversity” OR “landscape heterogeneity” OR “heterogeneous” OR “gene pool*" ) ) **(2103 DOCUMENTS)**

TITLE-ABS-KEY ( ( "NEGLECTED AND UNDERUTILISED" OR "NEGLECTED" OR "UNDERUTILISED" OR "ORPHAN" ) AND ( "CROP*" OR "SPECIES" OR "TREE*" ) AND ("Habitat" OR "invertebrates" OR "rodents" OR "biodiversity" OR "landscape diversity" OR "landscape heterogeneity" OR "heterogeneous" ) ) AND NOT vertebrate* OR animal* **(885 DOCUMENTS)**

**Maintenance of genetic diversity:**

TITLE-ABS-KEY ( ( "NEGLECTED AND UNDERUTILISED"  OR  "NEGLECTED"  OR  "UNDERUTILISED"  OR  "ORPHAN" )  AND  ( "CROP*"  OR  "SPECIES"  OR  "TREE*" )  AND  (“genetic diversity” OR “ecological diversity” OR OR “genetic storage” OR “agroecological diversity” OR “gene pool*" ) )  **(376 DOCUMENTS)**

TITLE-ABS-KEY ( ( "NEGLECTED AND UNDERUTILISED" OR "NEGLECTED" OR "UNDERUTILISED" OR "ORPHAN" ) AND ( "CROP*" OR "SPECIES" OR "TREE*" ) AND ("genetic diversity" OR "ecological diversity" OR "genetic storage" OR "agroecological diversity" OR "gene pool*" ) ) AND NOT vertebrate* OR animal*  **(245 DOCUMENTS)**

1. **CULTURAL SERVICES**

TITLE-ABS-KEY ( ( "NEGLECTED AND UNDERUTILISED"  OR  "NEGLECTED"  OR  "UNDERUTILISED"  OR  "ORPHAN" )  AND  ( "CROP*"  OR  "SPECIES"  OR  "TREE*" )  AND  ( " CULTURAL SERVICE*" ) )  **(1 DOCUMENTS)**

TITLE-ABS-KEY ( ( "NEGLECTED AND UNDERUTILISED"  OR  "NEGLECTED"  OR  "UNDERUTILISED"  OR  "ORPHAN" )  AND  ( "CROP*"  OR  "SPECIES"  OR  "TREE*" )  AND  ( " CULTURAL SERVICE*" ) ) AND NOT  vertebrate*  OR  animal* **(0 DOCUMENTS)**

**Eco- and cultural tourism:**

TITLE-ABS-KEY ( ( "NEGLECTED AND UNDERUTILISED"  OR  "NEGLECTED"  OR  "UNDERUTILISED"  OR  "ORPHAN" )  AND  ( "CROP*"  OR  "SPECIES"  OR  "TREE*" )  AND  ( " Eco-tourism” OR “cultural tourism” OR “cultural village*" ) ) **(1 DOCUMENT)**

TITLE-ABS-KEY ( ( "NEGLECTED AND UNDERUTILISED"  OR  "NEGLECTED"  OR  "UNDERUTILISED"  OR  "ORPHAN" )  AND  ( "CROP*"  OR  "SPECIES"  OR  "TREE*" )  AND  ( " Eco-tourism” OR “cultural tourism” OR “cultural village*" ) ) AND NOT  vertebrate*  OR  animal* **(0 DOCUMENTS)**

**Aesthetic, cultural heritage and knowledge and education:**

TITLE-ABS-KEY ( ( "NEGLECTED AND UNDERUTILISED"  OR  "NEGLECTED"  OR  "UNDERUTILISED"  OR  "ORPHAN" )  AND  ( "CROP*"  OR  "SPECIES"  OR  "TREE*" )  AND  ( " Aesthetic” OR “cultural heritage” OR “knowledge” OR “education” OR “beauty” OR “scenic” OR “peace” OR “creation” OR “indigenous knowledge systems” OR “tradition” OR “ceremony” OR “biodiversity” OR “inspiration”) ) **(1930 DOCUMENTS)**

TITLE-ABS-KEY ( ( "NEGLECTED AND UNDERUTILISED"  OR  "NEGLECTED"  OR  "UNDERUTILISED"  OR  "ORPHAN" )  AND  ( "CROP*"  OR  "SPECIES"  OR  "TREE*" )  AND  ( " Aesthetic” OR “cultural heritage” OR “knowledge” OR “education” OR “beauty” OR “scenic” OR “peace” OR “creation” OR “indigenous knowledge systems” OR “tradition” OR “ceremony” OR “biodiversity” OR “inspiration”) ) AND NOT  vertebrate*  OR  animal* **(959 DOCUMENTS)**

**Spiritual and Religious, sense of place:**

TITLE-ABS-KEY ( ( "NEGLECTED AND UNDERUTILISED"  OR  "NEGLECTED"  OR  "UNDERUTILISED"  OR  "ORPHAN" )  AND  ( "CROP*"  OR  "SPECIES"  OR  "TREE*" )  AND  ( " Spiritual” OR “religious” OR “ancestors” OR “prayer” OR “worship” OR “sense of place” OR “belonging” OR “sovereignty” OR “customs” OR “traditional knowledge" ) ) **(444 DOCUMENTS)**

TITLE-ABS-KEY ( ( "NEGLECTED AND UNDERUTILISED"  OR  "NEGLECTED"  OR  "UNDERUTILISED"  OR  "ORPHAN" )  AND  ( "CROP*"  OR  "SPECIES"  OR  "TREE*" )  AND  ( " Spiritual” OR “religious” OR “ancestors” OR “prayer” OR “worship” OR “sense of place” OR “belonging” OR “sovereignty” OR “customs” OR “traditional knowledge" ) ) AND NOT  vertebrate*  OR  animal* **(215 DOCUMENTS)**

1. **Supplementary Information 4**

## Results of literature search

**Table 4**: Title screening records

|  | **Keyword component** | **Articles retrieved** | **Eligible articles exported to Endnote** |
| --- | --- | --- | --- |
| **ECOSYSTEM SERVICES** | Ecosystem services 1 | 102 | 1 |
|  | Ecosystem services 2 **AND NOT** | 50 | 2 |
|  | Ecosystem services 3 | 2 | 0 |
| **PROVISIONING SERVICES** | Provisioning services: Food 1 | 2621 | 0 |
|  | Provisioning services: Food 2 **AND NOT** | 1653 | 0 |
|  | Raw materials 1 | 962 | 0 |
|  | Raw materials 2 **AND NOT** | 673 | 22 |
|  | Fresh water variation 1 (Flow regulation, water quality) | 79 | 0 |
|  | Fresh water variation 2 (water quantity, hydrological cycle) | 9 | 0 |
|  | Fresh water variation 3 (water purification, water flow) | 34 | 0 |
|  | Fresh water variation 4 (water regulation, freshwater, blue water) | 203 | 2 |
|  | Fresh water variation 5 (water regulation, surface water) | 56 | 2 |
|  | Medicinal 1 | 711 | 0 |
|  | Medicinal **AND NOT** | 24 | 1 |
|  | Medicinal variation (medicinal, traditional medicine) | 602 | 74 |
|  | Medicinal variation 2 (muthi*, curative, pharmaceutical) | 159 | 32 |
| **REGULATING SERVICES** | Local climate and air quality 1 | 118 | 0 |
|  | Local climate and air quality 2 **AND NOT** | 102 | 2 |
|  | Carbon sequestration and storage 1 | 792 | 4 |
|  | Carbon sequestration and storage 2 **AND NOT** | 569 | 3 |
|  | Moderation of extreme events 1 | 2798 | 2 |
|  | Moderation of extreme events 2 **AND NOT** | 1875 | 8 |
|  | Erosion prevention and soil fertility 1 | 2728 | 1 |
|  | Erosion prevention and soil fertility 2 **AND NOT** | 1823 | 6 |
|  | Pollination 1 | 114 | 1 |
|  | Pollination 2 **AND NOT** | 42 | 3 |
|  | Pollination variation 1 (Pollinate, pollinator) | 70 | 20 |
|  | Biological control variation 2 (bio efficacy, pest regulation, deterrents, pubescent) | 3 | 2 |
|  | Biological control 1 | 119 | 0 |
|  | Biological control 2 **AND NOT** | 52 | 0 |
|  | Biological control variation 1 (biological control, pest control) | 49 | 36 |
|  | Biological control variation 2 (bio efficacy, pest regulation, deterrents, pubescent) | 3 | 2 |
| **HABITAT AND SUPPORT** | Supporting | 4 | 0 |
|  | Supporting **AND NOT** | 2 | 0 |
|  | Habitat | 880 | 0 |
|  | Habitat **AND NOT** | 581 | 0 |
|  | Habitat of species | 2111 | 9 |
|  | Habitat of species **AND NOT** | 887 | 2 |
|  | Maintenance of genetic diversity | 376 | 40 |
|  | Maintenance of genetic diversity **AND NOT** | 245 | 20 |
| **CULTURAL SERVICES** | Cultural services | 1 | 0 |
|  | Eco-cultural tourism | 1 | 0 |
|  | Aesthetic, cultural heritage and knowledge education variation 1 | 1939 | 6 |
|  | Aesthetic, cultural heritage and knowledge education variation 2 **AND NOT** | 963 | 34 |
|  | Spiritual and religious sense of place variation | 446 | 11 |
|  | Spiritual and religious sense of place variation 2 **AND NOT** | 215 | 18 |
|  | **TOTAL** | **27848** | **366** |

During the initial search, the search terms yielded a total of 27848 hits. In cases where the search terms retrieved more than 500 hits, the results were sorted according to relevance to the objectives of the study. If no articles were relevant after screening the first 100, the rest of the articles for that specific search term were then deemed irrelevant. The titles were screened within the database and only articles that met the selection criteria were considered for further screening (Table 4 SI4). The titles, keywords and abstracts of 366 articles were then exported to Endnote reference manager to remove duplicates, which were 104 in total (see Tables 4 and 5 in Supplementary Information). Inclusion and exclusion criteria were then applied to the abstracts of the remaining 262 articles in Microsoft Excel, leaving 123 articles. The inclusion criteria for the search were articles referring to plants, and food systems while the exclusion criteria were articles that were not in English, books and book chapters, and articles that focussed on animals and invertebrates, and non-food crops. Book and book chapters were excluded due to the focus of the study on peer-reviewed literature

**Table 5:** Summary of articles retrieved

| **Keyword** | **Number of articles exported to Endnote** |
| --- | --- |
| Aesthetic, cultural heritage and knowledge | 40 |
| Biological control | 38 |
| Carbon sequestration and storage | 7 |
| Ecosystem services | 3 |
| Erosion prevention and soil fertility | 7 |
| Fresh water | 4 |
| Habitat and species | 11 |
| Local climate | 2 |
| Medicinal | 109 |
| Moderation of extreme events | 10 |
| Pollination | 24 |
| Provisioning services: food | 51 |
| Raw materials | 31 |
| Spiritual and religious sense of place | 29 |
| **Total** | **366** |
| **After removal of duplicates** | **262** |

1. **Supplementary Information 5**


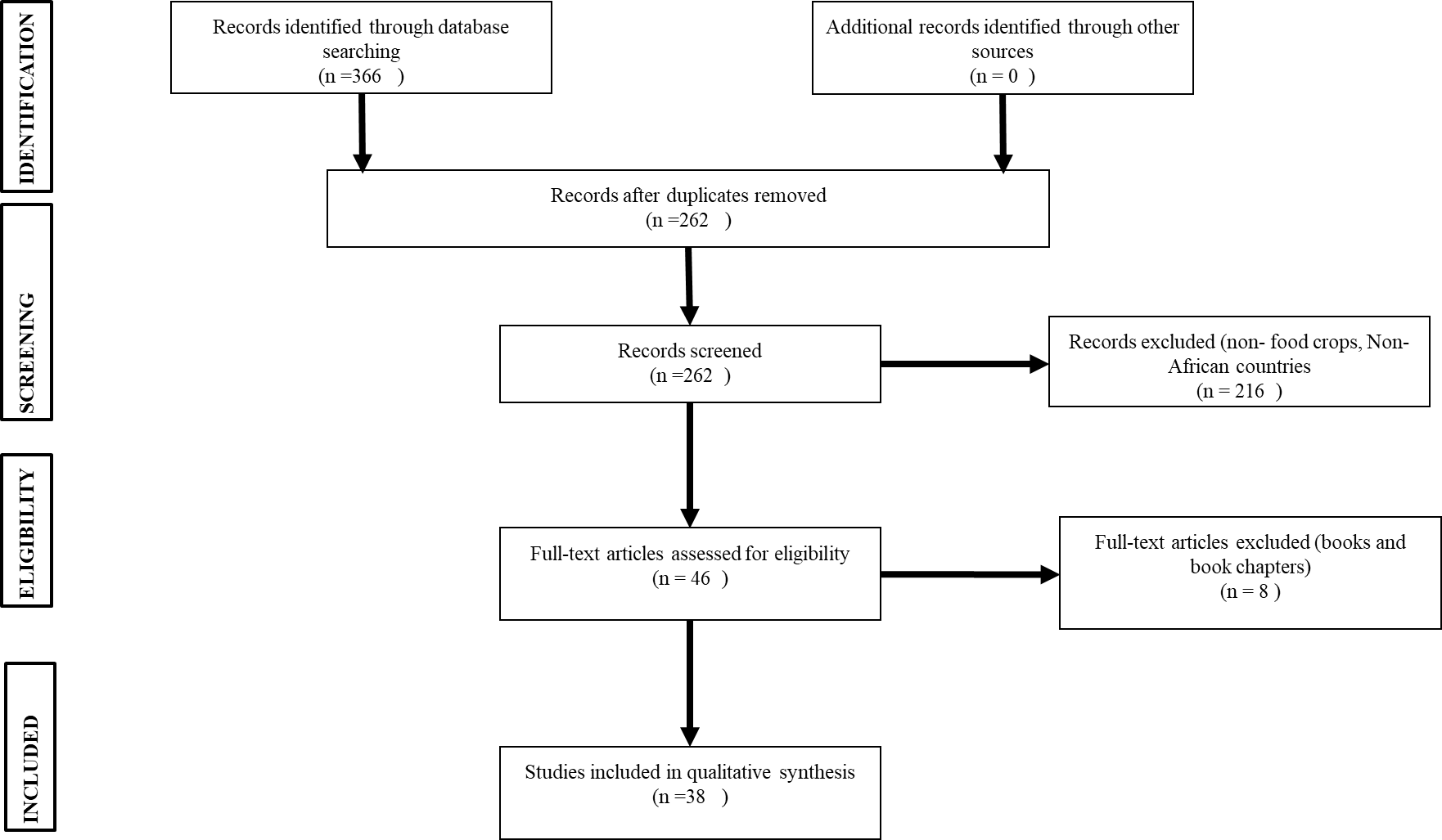

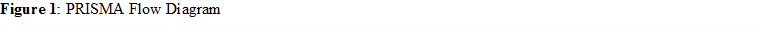


1. **Supplementary Information 6**

**Table 6:** List of studies included in the review

| **Authors (year)** | **Title of article** | **Article ID** |
| --- | --- | --- |
| Quartermain (2007) | Documentation of policies and strategies relating to underutilised species | 114 |
| Rudebjer et al. (2013) | Beyond commodity crops: Strengthening young scientists' capacity for research on underutilised species in sub-Saharan Africa | 112 |
| Azam-Ali (2007) | Agricultural diversification: The potential for underutilised crops in Africa's changing climates | 64 |
| Mokgolodi,et al. ( 2011) | Ziziphus mucronata: An underutilized traditional medicinal plant in Africa | 198 |
| Masondo et al. (2016) | Nutritional and pharmacological potential of the genus Ceratotheca - An underutilized leafy vegetable of Africa | 208 |
| Liu et al. (2011) | Cassava Genetic Transformation and its Application in Breeding | 31 |
| Hoeschle-Zeledon and Jaenicke (2009) | A strategic framework for research and development of underutilized plant species with special reference to Asia, the Pacific and Sub-Saharan Africa | 128 |
| Etèka et al. (2011) | Diversity, cultural practices and domestication of Sesamum radiatum Thonn. ex Hornem and Justicia tenella (Nees) T., two neglected and underutilised traditional leafy vegetables consumed in Benin | 137 |
| Tebkew et al. (2014) | Underutilized wild edible plants in the Chilga District, northwestern Ethiopia: Focus on wild woody plants | 105 |
| Hughes and Ebert (2013) | Research and development of underutilized plant species: The role of vegetables in assuring food and nutritional security | 164 |
| Nyadanu and Lowor (2014) | Promoting competitiveness of neglected and underutilized crop species: comparative analysis of nutritional composition of indigenous and exotic leafy and fruit vegetables in Ghana | 118 |
| Nyadanu et al. (2016) | Agro-biodiversity and challenges of on-farm conservation: the case of plant genetic resources of neglected and underutilized crop species in Ghana | 193 |
| Irungu et al. (2007) | Marketing of African leafy vegetables in Nairobi and its implications for on-farm conservation of biodiversity | 12 |
| Gotor and Irungu (2010) | The impact of Bioversity International's African Leafy Vegetables programme in Kenya | 135 |
| Onyango et al. (2013) | Current status on production and utilization of spider plant (Cleome gynandra L.) an underutilized leafy vegetable in Kenya | 190 |
| Gruca (2016) | Medicinal palms (Arecaceae) in Madagascar—undocumented or underutilized? | 165 |
| Adeoti et al. (2009) | Impact of gari consumption on the water resource of Nigeria | 69 |
| Pereira (2017) | Cassava bread in Nigeria: the potential of ‘orphan crop’ innovation for building more resilient food systems | 159 |
| Mabhaudhi et al. (2018) | Mainstreaming underutilized indigenous and traditional crops into food systems: A South African perspective | 6 |
| Allemann (2007) | Re-introduction of indigenous vegetables at the community level in South Africa | 22 |
| Mabhaudhi et al. (2013) | Response of taro (Colocasia esculenta L. Schott) landraces to varying water regimes under a rainshelter | 76 |
| Mabhaudhi et al. (2017) | Developing a roadmap for improving neglected and underutilized crops: A case study of South Africa | 123 |
| Mabhaudhi et al. (2017) | Status of underutilised crops in South Africa: Opportunities for developing research capacity | 163 |
| Weinberger (2007) | Are Indigenous vegetables underutilized crops? Some evidence from eastern Africa and South East Asia | 1 |
| Nitcheu Ngemakwe (2017) | Phytochemical and nutritional properties of underutilised fruits in the southern African region | 194 |
| Karunaratne et al. (2015) | Assessing the productivity and resource-use efficiency of underutilised crops: Towards an integrative system | 9 |
| Karunaratne et al. (2015) | Modelling the productivity of underutilised crops for climate resilience | 10 |
| Dinssa et al. (2015) | Traditional vegetables: Improvement and development in sub-Saharan Africa at AVRDC - The World Vegetable Center | 16 |
| Chivenge et al. (2015) | The potential role of neglected and underutilised crop species as future crops under water scarce conditions in Sub-Saharan Africa | 17 |
| Johns and Eyzaguirre (2006) | Linking biodiversity, diet and health in policy and practice | 62 |
| Towns and Shackleton (2018) | Towns, A. M. and C. Shackleton (2018). "Traditional, Indigenous, or Leafy? A Definition, Typology, and Way Forward for African Vegetables." Economic Botany 72(4): 461-477. | 2 |
| Kamnaratne et al. (2009) | Modelling the response of bambara groundnut: A key underutilised crop in agricultural systems | 124 |
| Campanaro et al. (2019) | DNA barcoding to promote social awareness and identity of neglected, underutilized plant species having valuable nutritional properties | 138 |
| Tele Ayenan and Ezin (2016) | Potential of Kersting's groundnut [Macrotyloma geocarpum (Harms) Maréchal & Baudet] and prospects for its promotion | 175 |
| Nyamupingidza, et al. (2013) | Using underutilised food crops to strengthen the food and nutritional status of an HIV-impacted community in the Uzumb-Maramba-Pfungwe district of Zimbabwe | 5 |

1. **Supplementary Information 7**

**Table 7:** List of NUS identified in articles

| **Crop type** | **Crop** | **Scientific name** |
| --- | --- | --- |
| **Cereals** | Pearl millet | *Pennisetum glaucum (L.) R.Br* |
|  | Buckwheat | *Fagopyrum esculentumMoench* |
|  | Finger millet | *Eleusine coracana* |
|  | Sorghum | Sorgum bicolar |
|  | African brown rice | *Oryza glaberrima* |
|  | Maize landrace | *Zea-mays L* |
|  | Fonio | *Digitaria exilis* |
|  | Quinoa | *Chenopodium quinoa* |
|  | Tef | *Eragrostis tef* |
| **Legumes** | Cowpea | *Vigna unguiculata* |
|  | Bambara groundnut | *Vigna subterranea L. Verdc* |
|  | Pigeonpea | *Cajanus Cajan* |
|  | African yam bean | *Sphenostylus stenocarpa* |
|  | Kersting`s groundnut | *Macrotyloma geocarpum* |
|  | Sunhemp | *Crotalaria juncea* |
|  | Mung bean | *Vigna radiata* |
|  | Andean Lupin | *Lupinus mutabilis* |
|  | Lablab bean | *Lablab purpueus* |
|  | Sword bean | *Canavalia gladiata* |
|  | Marama bean | *Tylosema esculentum* |
|  | Velvet bean | *Mucuna pruriens* |
| **Roots and Tubers** | Cassava | *Manihot esculenta* (123 p 6) |
|  | Taro | *Calocasia esculenta* |
|  | Air potato | *Dioscorea bulbifera* |
|  | Sweet potato | *Ipomea batatus (L.) Lam* |
|  | Parsnip | *Pastinaca sativa* |
|  | Bitter yam | *Dioscorea dumetorum* |
|  | Arrow roots | *Colocasia spp.* |
|  | Yams | *Dioscorea spp.* |
|  | Cocoyam | *Xanthosoma spp.* |
| **Leafy Vegetables, herbs and vegetables** | Pigweed | *Amarantha sp* |
|  | Eggplant | *Solanum spp* |
|  | Gourd | *Cucurbita spp.* |
|  | Autumn squash | *cucurbita maxima* |
|  | Squash | *Cucurbita moschata* |
|  | Summer squash | *Cucurbita pepo* |
|  | Malabar gourd | *Cucurbita facifolia* |
|  | Wild watermelon | *Citrullus lanatus* |
|  |  | *Coccinia adoensis* |
|  | Horned melon | *Cucumis metuliferus* |
|  | South African Spiny cucumber | *Cucumis zeyheri* |
|  | African cucumber | *Momordica balsamina* |
|  | Spider plant | *Cleome gynandra L* |
|  | Okra | *Abelmoschus esculentus L Moench* |
|  | Okra | *Hibiscus esculenta* |
|  | African nightshade | *Solunum scabrum* |
|  | Eggplant | *Solunum aethiopicum L* |
|  | African eggplant | *Solunum macrocarpon L* |
|  | Pigweed | *Amaranthus cruentus L* |
|  | Pigweed | *Amaranthus dubius Mart. Ex Thell* |
|  | Pigweed | *Amaranthus hybridus L* |
|  | Pigweed | *Amaranthus blitum* |
|  | Pigweed | *Amaranthus caudatus* |
|  | Pigweed | *Amaranthus hypochondriacus* |
|  | Pigweed | *Amaranthus palmeri* |
|  | Pigweed | *Amaranthus quitensis* |
|  | Pigweed | *Amaranthus tricolar* |
|  | Pigweed | *Amaranthus viridis* |
|  | Pigweed | *Amaranthus retroflexus* |
|  | Pigweed | *Amaranthus spinosus* |
|  | Pigweed | *Amaranthus thunbergii* |
|  | Blackjack | *Bidens pilosa* |
|  | Living stone potato | *Plectranthus eculentus* |
|  | Eggplant | *Solanum macrocarpa L* |
|  | Eggplant | *Solanum melongena* |
|  | Cañihua | *Chenopodium pallidicaule* |
|  | Eggplant | *Solunum aethiopicum L* |
|  | Chard | *Beta vulgaris* |
|  | Melon | *Cucumis melo subsp agrestis* |
|  | Worowo | *Solanecio biafrae* |
|  | Cockscomb | *Celosia argentea* |
|  | Pumpkin | *Cucurbita spp.* |
|  | Slippery cabbage | *Abelmoschus Manihot* |
|  | Water spinach | *Ipomea aquatica* |
|  | Hyacinth bean | *Lablab purpueus* |
|  | Jute mallow | *Corchorus olitorius* |
|  |  | *Crotalaria ochroleuca* |
|  |  | *Crotalaria brevidens* |
|  | Bottle gourd | *Lagenaria siceraria* |
|  | Ridged gourd | *Luffa acutangula* |
|  | Cucumber | *Cucumis sativa* |
|  | Ethiopian mustard | *Brassica carinata* |
|  | Sesame | *Sesamum radiatum* |
|  | Sesame | *sesamum indicum* |
|  |  | *Justicia tenella* |
|  | Sesame | *sesamum indicum* |
|  | Elephant ear | *Xanthosoma sagittifolium* |
|  | Prickly nightshade | *Solunum torvum* |
|  | False sesame | *Ceratotheca sesamoides* |
|  | Wild foxglove | *Ceratotheca triloba* |
|  | Roselle | *Hibiscus sabdariffa* |
|  | Wild mustard | *Brassica juncea* |
|  | Wild mustard | *Brassica niagra* |
|  | Fluted pumpkin | *Telfaria occidentalis* |
|  | Sicklepod | *Cassia obtusifolia* |
|  | Yoruban bologi | *Crassocephalum rubens* |
|  | Chinese cabbage | *Brassica rapa* |
|  | Sun-berry | *Solanum retroflexum* |
|  | Gallant soldier | *Galinsoga parviflora* |
|  | Yellow justica | *Justicia flava* |
|  | Sticky gooseberry- move shrub | *Physalis viscose* |
|  | Giant bell flower | *Wahlenbergia undulata* |
|  | Purslane | *Portulaca aleracea* |
|  | Stalks talk | *Oxygonum sinuatum* |
|  | Devil`s thorn | *Tribulus terrestris* |
|  | Spindle pod | *Chenopodium album* |
|  | Black nightshade | *solanum modiflorum* |
| **Fruits, from trees and shrubs** | Baobab tree | *Adansonia digitata* |
|  | Moringa | *Moringa olefeira L* |
|  | Breadfruit | *Artocarpus altilis* |
|  | Breadnut | *Artocarpus camansi* |
|  | Buruti- Palm fruit | *Mauritia flexuosa L* |
|  | Buffalo thorn | *Ziziphus mucronata* |
|  | coconut tree | *Cocos nucifera L* |
|  | Raffia Palm | *Raphia fariniferi (Gaertn.) Hyl.* |
|  | Areca Palm | *Dypsis spp* |
|  | Evergreen palm tree | *Dypsis fibrosa (CH. Wright) Beentjie* |
|  | Solitary-stemmed evergreen palm | *Dypsis pinnatifrons Mart* |
|  | Single-stemmed, evergreen palm tree | *Ravenea sambiranensis Jum* |
|  | Majestic palm | *Ravenea spp* |
|  | General palm name | *Dypsis andrianatonga* |
|  | Palm-heart bitter | *Dypsis canaliculata (Jum.) Beentjie* |
|  | Vonitra | *Dypsis crinita (Jum. & H.Perrier* |
|  | General palm | *Dypsis lastelliana* |
|  |  | *Dypsis louvelii Jum* |
|  | Madagascar palm | *Dypsis madagascariensis* |
|  | Solitary palm | *Dypsis tsaravoasira* |
|  | Golden cane palm | *Dypsis viridis* |
|  | African oil palm | *Elaeis guineensis Jacq* |
|  | Lala palm | *Hyphaene coriacea Gaertn* |
|  |  | *Masoala kona Beentje* |
|  | Akoraka | *Revenea glauca Jum. H.Perrier* |
|  | African medlar | *Vangeria infausta* |
|  | Monkey orange | *Strychnos spinosa* |
|  | Spinkey monkey | *Strychnos cocculoides* |
|  | Common jujube | *Ziziphus mauritiana* |
|  | Wild loquat | *Uapaca kirkiana* |
|  | Chocolate berry | *Vitex payos* |
|  | Mobola plum | *Parinari curatellifolia* |
|  | Kei apple | *Dovyalis cafra* |
|  | Sour plum | *Ximenia americana* |
|  | Cape gooseberry | *Physalis peruviana* |
|  | Marula | *Sclerocarya birrea* |
|  | Prickly pear | *Opuntia spp.* |
|  | Mexican wild apple | *Uapaca spp.* |
|  | Vine spinach | *Basella alba* |
|  | Red milk wood | *Mimusops zeyheri* |
|  | Bear's breeches | Acanthus senni |
|  | Thron tree | *Balanites aegyptiaca* |
|  | Penny bun | *Boletus edulis* |
|  | Jew's mallow- move to leafy veg | *Cochorus olitorius* |
|  | Bush plum | *Carissa Spinarum* |
|  | *ARABIC* inderab | *Cordia Africana* |
|  | Kalahari Christmas tree | *Dichostachys cinerea* |
|  | Purple yam | *Dioscorea prahensilis* |
|  | Giant diospyros | *Diospyros absyssinica* |
|  | African ebony | *Diospyros mesiliformis* |
|  | Abyssinian Gooseberry | *Dovyalis absyssinica* |
|  | Broom cluster fig | *Ficus sur Forssk* |
|  | Sycamore fig | *Ficus sycomorus* |
|  | False cape fig | *Ficus vallis-choudae* |
|  |  | *Ficus vasta Forssk* |
|  | White berry-bush | *Flueggea virosa Guill.* |
|  | Wild gardenia | *Gardenia ternifolia* |
|  | Flame lily | *Gloriosa superba* |
|  | Deccan hemp | *Hibiscus cannabinus* |
|  | Manding-Bambara | *Mimusops kummel* |
|  | Black mulberry | *Morusmeso zygia* |
|  | Cheesewood | *Pittosporum viridiflorum* |
|  |  | *Rhus glutinosa* |
|  | Abyssinian rose | *Rosa abyssinica* |
|  | Bungo | *Saba comorensis* |
|  | Dropseed | *Sporobolus Africanus* |
|  | Woodland waterberry | *Syzygium guineese* |
|  | Tamarind | *Tamarindus indica* |
|  | Large jujube | *Ziziphus abyssinica* |
|  | Christ's thorn jujube | *Ziziphus spina-christi Willd.* |
|  | Fennel | *Coriandrum sativum* |
|  |  | *Foeniculum vulgare* |
|  |  | *Heteromorpha trifoliata* |
|  | African wormwood- leafy veg | *Pimpinella anisum* |
|  |  | *Artemisia Afra Wild* |
|  | Mulavatwa (Kamba) | *Blumea brevipes* |
|  | Chamomile- move to leafy veg | *Helichrysum odoratissimum* |
|  |  | *Matricacaria chamomilla* |
|  |  | *Microglosa pyrrhopappa* |
|  | Cheptogomda | *Agnew var pyrrhopappa* |
|  |  | *Sphaeranthus bullatus Matt f* |
|  | Kasha (Pare) | *Sphaeranthus cyathuloides* |
|  | Cheptogomda | *Psidia punculata* |
|  | Mariricua | *Sphaeranthus suaveolens* |
|  | Olibanum | *Tarconanthus camphoratus* |
|  | Camphor bush | *Boswellia neglecta S.Moore* |
|  | Corkwood | *Commiphora holtziana* |
|  | African myrrh | *Commiphora myrrha* |
|  | Forest fever-berry | *Croton sylvaticus* |
|  | Euphorbia | *Synadenium compactum* |
|  | African basil | *Ocimum gratissium* |
|  | Holy basil | *Ocimum keniense* |
|  | Camphor basil | *Ocimum kilimandscharicum* |
|  | Coleus | *Plectranthus amboinicus* |
|  | Blue Spur Flower | *Plectranthus barbatus* |
|  |  | *Plectranthus marrubioides* |
|  |  | *Plectranthus sylvestris* |
|  | Angolan blue | *Plectranthus tenuiflorus* |
|  | Highveld Lemon Savory | *Saturea biflora* |
|  | Blue gum | *Eucalyptus globulus* |
|  | Least snout-bean | *Rynchosia minima* |
|  | Citronella grass | *Cymbogon citratus* |
|  | citronella grass | *Cymbogon nardus* |
|  | East African Sandal wood | *Clausena anisata* |
|  | Sandalwood | *Osyris lanceolata* |
|  | Lemon Verbena | *Lippia carviodora* |
|  |  | *Lippia dauensis* |
|  |  | *Lippia grandifolia* |
|  | Fever tea | *Lippia javanica* |
|  |  | *Lippia somalensis* |
|  | Mexican oregano | *Lippia ukambensis* |

Figure 2: Neglected and underutilised crop types

# Supplementary Information 8

**Table 8**: Number of articles referring to ecosystem services

|  | **Ecosystem services** | **References/Article IDs** | **Number of articles** |
| --- | --- | --- | --- |
| PROVISIONING | Food and nutrition | 1, 2, 5, 9, 10, 12, 16, 17, 22, 31, 62, 64, 69, 76, 105, 112, 114, 118, 123, 124, 137, 138, 163, 164, 175, 190, 194, 198, 208 | 29 |
|  | Raw material | 5, 17, 31, 64, 105, 118, 165, 175, 194, 198, 208 | 11 |
|  | Medicine: traditional or pharmaceutical | 2, 5, 6, 16, 17, 62, 105, 118, 123, 164, 165, 175, 190, 194, 198, 208 | 16 |
|  | Feed and fodder | 10, 17, 31, 105, 175, 194, 198, 208 | 8 |
| REGULATING | Biological control |  | 0 |
|  | Pollination | 17, 137, 198 | 3 |
|  | Climate | 2, 9, 10, 16, 17, 31, 64, 112, 123, 163, 164, 175, 208 | 13 |
|  | Carbon Sequestration/Storage |  | 0 |
|  | Erosion prevention and soil fertility | 17, 105, 175 | 3 |
|  | Moderation of extreme events | 9, 10, 17, 195, 163, 164 | 6 |
|  | Water regulation | 9, 17, 76, 105, 138, 163, 164 | 7 |
|  | Habitat for species |  | 0 |
| HABITAT AND SUPPORT | Maintenance of genetic diversity | 2, 9, 17, 112, 114, 118, 123, 135, 138, 163, 164, 175, 193, 208 | 14 |
| CULTURAL | Local knowledge | 1, 2, 10, 17, 62, 64, 112, 137, 138, 163, 165, 198 | 12 |
|  | Cultural heritage | 2, 12, 22, 62, 137, 175, 198 | 7 |
|  | Spiritual religious sense of place | 165, 175, 197, 198 | 4 |
|  | Eco- and cultural tourism | 105, 175, 198 | 3 |
